# Emergence of monopoly–Copper exchange networks during the Late Bronze Age in the western and central Balkans

**DOI:** 10.1371/journal.pone.0263823

**Published:** 2022-03-11

**Authors:** Mario Gavranović, Mathias Mehofer, Aleksandar Kapuran, Jovan Koledin, Jovan Mitrović, Aleksandra Papazovska, Andrijana Pravidur, Aca Đorđević, Dragan Jacanović

**Affiliations:** 1 Austrian Archaeological Institute, Austrian Academy of Science, Vienna, Austria; 2 VIAS-Vienna Institute for Archaeological Science, University Vienna, Vienna, Austria; 3 Institute of Archaeology, Belgrade, Serbia; 4 Museum of Vojvodina, Novi Sad, Serbia; 5 Department of Archaeology, National Museum Belgrade, Belgrade, Serbia; 6 Archaeological Museum of North Macedonia, Skopje, North Macedonia; 7 National Museum of Bosnia and Herzegovina, Sarajevo, Bosnia and Herzegovina; 8 National Museum in Požarevac, Požarevac, Serbia; University at Buffalo - The State University of New York, UNITED STATES

## Abstract

In this paper we present the first results of an interdisciplinary research project focused on Late Bronze Age metallurgy in the western and central Balkans. The comprehensive chemical and lead isotope analysis, and a strict consideration of archaeological criteria, has provided a deeper insight into supra regional metal exchange networks between the 14^th^ and 9^th^ century BC in this part of Europe. Particularly interesting and surprising are results regarding the provenance of raw materials for copper production, which have a chemical composition and lead isotope ratios that closely correspond to ore deposits in the southern Alps (North Italy). Based on the examination of 57 objects of different functions, chronology and distribution, it becomes apparent that copper from the southern Alps was almost an omnipresent raw material in the territories of the western and central Balkans with only a few finds from North Macedonia to indicate alternative sources. The analyses demonstrate that the reuse of fahlore-based copper is attested for the first time in the regions under study. The remarkable fact that other archaeological parameters do not indicate such an intensive connection between the Balkan area and Northern Italy raises a number of questions. The sustained and long-lasting networks of raw material procurement stand in contrast to the expected cultural interaction between metal producing and metal consuming prehistoric societies. The results of this work also highlight the currently underestimated role of the southern Alps as one of the main copper producing areas in Bronze Age Europe, and demonstrate for the first time that the region of western and central Balkans was one of the major recipients.

## Introduction

In contrast to numerous studies that have pointed out the pivotal role of the Balkans for the start of copper metallurgy as early as the 6^th^ millennium BC, our knowledge about the later development in this region is still limited by the lack of analytical data. Especially intriguing are the stages of the Bronze Age between the 14^th^ and the 9^th^ centuries BC, when the archaeological record suggests a peak in metal production and a significant increase of domestic metallurgy [1–4]. For this reason, Late Bronze Age metal finds from the Balkans have been the focus of several archeometallurgical projects [5–9], but the question of copper provenance has remained unanswered. A number of hypotheses about the use of abundant domestic copper ore sources during the Bronze Age have been put forward, but the evidence remains scarce [10, 11]. Clear traces of prehistoric mining and smelting have not been identified in any of the potentially promising ore rich regions. The only exception is the area around the city of Bor in eastern Serbia where recent investigations confirmed the engagement of local communities in copper ore exploitation and copper production during the Early and Middle Bronze Age between 19^th^ and 16^th^ centuries BC [12, 13]. However, for reasons still unclear, copper production in eastern Serbia declined around 1600 BC. Therefore, the raw material from this region could almost certainly be excluded as a source for the objects discussed here.

Bearing in mind the state of research, our primary goal was to collect and evaluate a sufficient amount of data that could provide new insights into the provenance of copper during the Late Bronze Age or between the 14^th^ and 9^th^ century BC in the western and central Balkans. In geographical terms, the western Balkans includes the region of the Dinaric Alps in modern Slovenia, Croatia and Bosnia-Herzegovina between the Sava River and Adriatic Sea, while the central Balkans are framed by the Danube River and include the regions along the Morava and Vardar valleys in today’s Serbia and North Macedonia. The chronological scope of nearly 500 years enables us also to track diachronic changes and developments throughout the centuries being discussed. However, several important aspects need to be considered. The intermediary geographic position of the study area between central Europe and the Aegean-Mediterranean world, highlighted by the presence of metal finds characteristic for both regions must be taken into account. At the beginning of the Late Bronze Age in the 13^th^ and 12^th^ centuries BC (BA D–Ha A1), most of the metal repertoire shows a clear affiliation with the Urnfield culture circle with prevalence of bronze objects from central Europe [14–16]. In order to gain a better insight into metal circulation during this time, we also included several copper ingots in the analysis as they represent an intermediary step between raw materials and finished objects.

The following period of the 11^th^ and 10^th^ centuries BC (Ha B1) is characterized by the emergence of regional bronze object types (swords, axes, pins, spear heads, bracelets, sickles) with a distribution across different landscapes of the Balkans and in the Carpathian Basin [16, 17]. Finally, the range of metal objects from the 9^th^ century BC (Ha B3) includes almost exclusively locally distributed weapons and jewelry, indicating that their production is to be sought in casting workshops situated in the same territories [2, 18].

## Questions and objectives

Given the archaeological, typological and chronological diversity of our samples, the first question that arises is if and how this diversity is reflected in the archaeometallurgical data. Through the combination of the newly obtained analytic results (chemical composition, lead isotope analysis) and the results of the archaeological research, our main intention in this paper is to provide a first diachronic overview of previously unknown Late Bronze Age copper supply networks in the western and central Balkans. Therefore, a crucial part of our analyses was to narrow down the possible sources of copper used in bronze production as the procurement of raw material implies direct or indirect contact and exchange. Even if one takes into account the limits of LIA in terms of association or rather dissociation between objects and ore deposits, the results presented below suggest the existence of a copper procurement network that was not considered or even assumed in previous research.

The essential questions for our research can be formulated as follows:

- Is there any analytical evidence that would speak for the exploitation of local copper ore sources during the Late Bronze Age?
- Which metal exchange networks can be reconstructed and described for the regions under study, and which are the most probable raw material sources for domestic metal workshops?
- Is the reuse of fahlore-based copper during the Late Bronze Age recognizable in the archaeometallurgical record? Does the metal supply in the Balkans follow the general trends during the Late Bronze Age as observed in neighboring regions [19–22]?
- Which alloying practices can be described in a chronological and typological perspective?

## Cultural context and dating

In the periods prior to LBA, the copper from the deposits in Eastern Serbia and Bulgaria had a dominant role in the regional supply and exchange networks [22–26]. As a number of studies have demonstrated, during the Chalcolithic, the copper from the East Serbian Copper Belt between the cities of Majdanpek and Bor was not only the main source of raw material for the Balkans but was obviously also traded into central Europe and even further to the north [27, 28]. The available data for the Early Bronze Age or 3^rd^ millennium BC indicate that the copper ore sources in the Balkans still played a significant role in the raw material procurement [29–31]. In the first half of the 2^nd^ millennium BC, local Bronze Age communities in eastern Serbia near the city of Bor began to exploit deposits in the vicinity by smelting of sulfidic copper ores [13]. Our previous analyses demonstrated that the copper from the Bor area circulated on a regional scale between 1900 and 1500 BC as some of the typical bronze objects from the Balkans were obviously made of this material [13, 30]. The production of copper in eastern Serbia ceased around 1600 BC and metal from other territories, including the Alpine region, started to appear in the Balkans and in the neighboring area of the Carpathian Basin [13, 32]. At the same time, copper from the eastern Mediterranean (Cyprus) entered the eastern Balkans (Bulgaria and southeast Romania) in the form of oxhide ingots, also underlining the connection of this area with the Aegean copper networks [33].

To date, archaeometallurgical studies looking at Late Bronze Age metal objects from the western and central Balkans have provided a solid overview of their chemical composition and indicated several general changes that occurred during the period between the 14^th^ and the 9^th^ centuries BC, yet tangible evidences for the sources of the copper raw materials are still lacking. The large data sets from Slovenia pointed to an increase in impurities (arsenic, nickel and antimony) in the metals from 11^th^ and 10^th^ centuries BC (Ha B in terms of Central European chronology) as compared to finds from 13^th^ and 12^th^ centuries BC or Ha A period [5, 34]. This resulted in the hypothesis that the difference in the chemical composition may be caused by the general change of ore sources, but lead isotope analyses that could confirm or disprove this possibility were not conducted. Additionally, the analyses of objects from Slovenia demonstrated a substantial increase of lead in the finds of Ha B period (mean 4.28%, compared to 2.84% from Ha A), indicating deliberate alloying of lead with copper.

The chemical analyses of objects from Bosnia and Herzegovina displayed similar tendencies to a certain extent [6, 8]. The metal finds from Ha B (11^th^–9^th^ century BC) regularly contained copper with higher concentrations of silver than in previous periods, while the value of other trace elements tends to vary regardless of chronology. One noteworthy exception is a group of objects from the 9^th^ century BC that display a striking consistency in terms of their chemical composition with higher concentrations of silver, nickel and antimony. In contrast to data from Slovenia, the metals analyzed from Bosnia-Herzegovina did not suggest any additional mixing of lead.

Apart from Slovenia and Bosnia-Herzegovina, the results of the archaeometallurgical analyses are also available for only a few finds from Serbia where the focus is the tin values [9, 35]. However, no general trend in terms of chronology and copper alloy mixtures are currently observable for the finds from the territory of today’s Serbia due to the limited quantity of data.

The results obtained in these past studies also make clear that there is no general decrease of tin in the Ha B period when compared to older finds from Ha A. The tin percentage seems to relate to the function of the object rather than chronology. In particular, the large data set from Slovenia (with almost 600 analyzed finds) reveals a clear correlation between object categories and tin content, for instance with spearheads and swords having consistently more tin than axes and sickles [34]. Our dataset confirms the results from Slovenia, with some specific variations that will be discussed below.

In addition to studies of the chemical composition, evidence of workshops engaged in metal casting and alloying is another important factor in better understanding metallurgy in the western and central Balkans during the Late Bronze Age. Based on the quantity and distribution of casting equipment (moulds, crucibles, cores), the local metal production seems to have greatly increased between the 11^th^ and 9^th^ centuries BC, with a concentration of casting sites in the Dinaric Alps between the Sava River and the Adriatic [3, 36]. The location of production sites such as Varvara, Donja Dolina, Ripač and Pivnica in the relative vicinity of copper ore deposits in Bosnia has led to the assumption that the rise of the new foundries and the position of nearby copper sources might be related, yet there is no analytic data to confirm this [10, 37]. Judging by the repertoire of the cast metal objects, the aforementioned workshops were part of a regional distribution network that spanned the area between the southern Carpathian Basin and the Adriatic coast [3, 36]. Whilst the existence of domestic workshops at the end of second millennium BC undeniably implies increased demand for raw materials, supply networks that were operating in the context of growing metal production in the area remain unknown. In terms of general connectivity and exchange networks, the close ties between the study area and the Carpathian Basin are clearly recognizable in all aspects of the archaeological record, especially during the time between the 14^th^ and 12^th^ centuries BC, including the distribution of widely spread “international” bronze types [2, 4, 14, 38–40]. The finds of Mycenaean rapiers from North Macedonia and Kosovo suggest that the Late Bronze Age communities in this part of the Balkans were partly included in the distribution networks of the Aegean world, which is not surprising given the geographical position [41–43]. From the 12^th^ century BC onwards, several new tendencies can be observed with regard to general connectivity networks. The relation with the Carpathian Basin and central Europe (Urnfield culture) seems to be less intensive with the emergence and establishment of several local groups with a specific spectrum of pottery, metal items and with distinct burial practices, in particular within the mountainous zone of the Dinaric Alps [11, 44–46]. The finds from the Adriatic coast and its hinterland, especially the metal items, also indicate the increased exchange with the Apennine Peninsula, via the Adriatic Sea as a mutual communication area [47–49]. According to the available metal finds of personal equipment (e.g. fibulae, swords), the connections between parts of the western Balkans and Italy most probably involved occasional movement of individuals, while traces of any kind of major cultural transfer that would have an impact on e. g. pottery or burial customs are not existent [50, 51]. Despite these obvious influences from neighboring regions, most of the local groups in both the western and central Balkans between the 11^th^ and 9^th^ centuries BC have a distinct, idiosyncratic archaeological record, which is most clearly expressed in the creation of specific local metal object types, some of which are included in the here presented analysis.

## Materials

**Fig 1:**
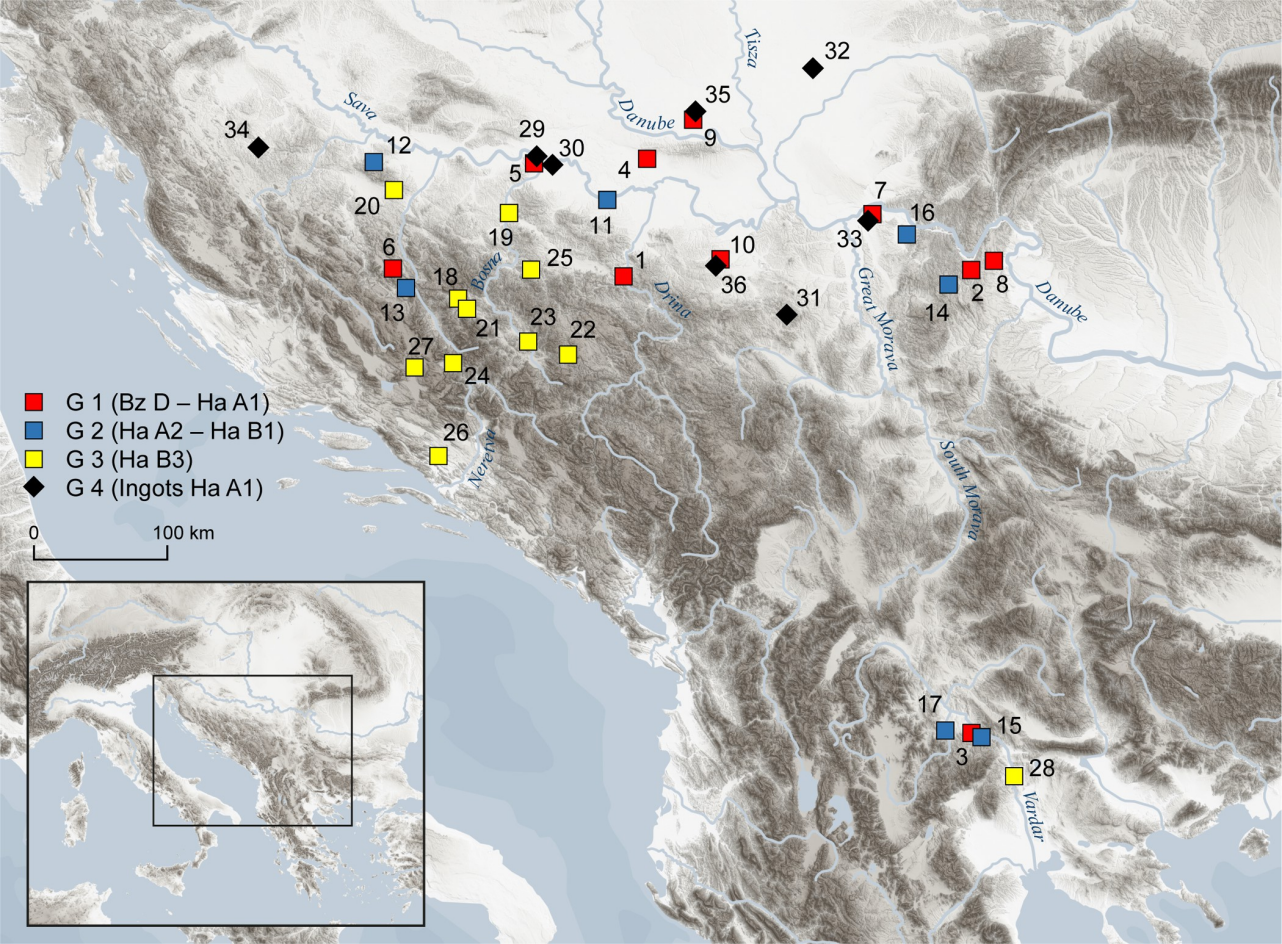
Map of the sites included in the presented analyses. 1: Radaljska Ada; 2: Topolnica; 3: Mali Dol; 4: Privina Glava; 5: Novigrad; 6: Antonići; 7: Kličevac-Rastovača; 8: Urovica; 9: Futog; 10: Trlić; 11. Brezovo Polje; 12: Vojskova; 13: Drenov Do; 14: Leskovo; 15: Demir Kapija; 16: Vojilovo; 17: Sivec; 18: Travnik; 19: Grapska; 20: Ivanjska; 21: Veliki Mošunj; 22: Gradac-Sokolac; 23. Ilijaš; 24: Prozor; 25: Hrge-Lučica; 25: Vitina-Otok; 27: Pakline; 28: Milci; 29: Novigrad; 30: Topolovaca Bregovi; 31: Rudnik; 32: Hetin; 33. Kličevac-Rastovača; 34: Podzvid-Šumatac; 35: Futog; 36: Trlić (Map made by I. Petschko, source digital elevation model: NASA JPL (2013). *NASA Shuttle Radar Topography Mission Global 3 arc second* [Data set]. NASA EOSDIS Land Processes DAAC. https://doi.org/10.5067/MEaSUREs/SRTM/SRTMGL3.003; vector data of water bodies: Natural Earth https://www.naturalearthdata.com (public domain).

From a total of 560 with ED-XRF analyzed metal objects dated to different stages of the Bronze Age, 221 of them were additionally analyzed with LIA (EBA/MBA: 26; LBA:195). Out of our LBA set with ED- XRF and LIA data, we now discuss a representative selection of 45 metal objects and twelve ingots (Table 1). For one of the ingots with two layers (VIAS lab. no. BelM 474 and 475) we have two results at hand, resulting in total number of 58 analyses (Table 1). Based on their archaeological characteristics (context, dating, form, function and distribution), the chosen finds are an illustrative sample set for regional and inter-regional distribution tendencies as well as contact networks in the area during the LBA. Regarding the distribution of each of the specific archaeological types, it is clear that occurrence in certain regions alone does not necessarily correlate with the place of production nor that the typology of the object and material composition are necessarily related to each other. Nevertheless, the limited distribution of distinct objects in a particular environment increases the possibility that the items were made in workshops situated not too far away. The primary intention of our research is to elucidate which mining and smelting regions supplied the local workshops. In the case of widely distributed objects, the place of production can only be speculated. The probability that the deposition of these objects across the western and central Balkans is an outcome of general mobility should certainly be considered. In order to verify possible differences, we deliberately included objects not typical for the study area within the selection. In a next step, the selected finds are divided into three chronological groups (Group 1, 2, and 3) with the aim of evaluating the results of the archaeometallurgical analyses in a diachronic perspective (Fig 1). The three groups broadly correspond with the stages of the Urnfield period BA D–Ha A1 (14^th^–12^th^ centuries BC) = Group 1, Ha A2–Ha B1 (11^th^–10^th^ centuries BC) = Group 2 and Ha B3 (9^th^ century BC) = Group 3. Each group contains objects of regional and interregional distribution. The twelve copper ingots from stage Ha A1 form an additional group of samples (Group 4).

**Table 1:** Sampled metal objects.

Site	Object	Context	Group	Inv./ Mus. no.	Museum/ depository	Location/Country	Distribution/ provenance	Bibliography	VIAS lab. no.	CEZA Mannheim lab. no.
Antonići	Flange hilted sword, type Reutlingen	Singular deposition	1	19914	National Museum Sarajevo	BiH	Central Europe, Carpathian Basin, SO Europe	Harding 1995, 35	SJLM 62	MA-186728
Novigrad	Spearhead	Hoard	1	32790	National Museum Sarajevo	BiH	Carpathian Basin, SO Europe	König 2004, 212	SJLM 38	MA-186715
Radaljska Ada	Flange hilted sword, type Aranyos	River find	1	83 = 510	National Museum Sarajevo	BiH	Carpathian Basin, SO Europe	Harding 1995, 28	SJLM 60	MA-186726
Futog	Spearhead	Hoard	1	3332	Vojvodina Museum Novi Sad	SRB	Carpathian Basin, SO Europe	Vasić 2015, 54	VoNSM 170	MA-186783
Futog	Sickle, type Uioara 1	Hoard	1	3343	Vojvodina Museum Novi Sad	SRB	Central Europe, Carpathian Basin, SO Europe	Vasić 1994, 27	VoNSM 177	MA-196158
Futog	Pin with ripped flattened head	Hoard	1	3455	Vojvodina Museum Novi Sad	SRB	Carpathian Basin, SO Europe	Vasić 2003, 71	VoNSM 183	MA-186786
Topolnica	Socketed axe	Hoard	1	167	Negotin Museum	SRB	Transylvania, Lower Danube	Jovanović 1975, 83	NegM9	MA-196018
Kličevac-Rastovača	Flange hilted sword, type Reutlingen, variant Staro Topolje	Hoard	1	2131	Požarevac Museum	SRB	Western and central Balkans	Jacanović 2000, 36	PozM 32	MA-186760
Topolnica	Flange hilted sword, type Reutlingen, variant Genf	Hoard	1	1759	Negotin Museum	SRB	Central Europe and Balkans	Harding 1995, 40	NegM 1	MA-186739
Topolnica	Full hilted sword, type Riegsee	Hoard	1	1757	Negotin Museum	SRB	Central Europe and Carpathian basin	Harding 1995, 71	NegM 6	MA-186742
Topolnica	Decorated armlet	Hoard	1	1755	Negotin Museum	SRB	Carpathian Basin	Jovanović 1975, 82	NegM 17	MA-186745
Trlić	Flange hilted sword, type Reutlingen, local variant	Hoard	1	14457	National Museum Belgrade	SRB	Western and central Balkans	Harding 1995, 43	BelM 408	MA-196286
Mali Dol	Knife	Grave	1	22669	National Museum Skopje	MKD	Central Balkans	Papazovska 2019, 125	SkoM 276	MA-196219
Urovica	Socketed axe	Hoard	1	167	Negotin Museum	MKD	Transylvania, Lower Danube	Srejović 1975, 97	NegM24	MA-186747
Privina Glava	Socketed axe	Hoard	1	2061	National Museum Belgrade	MKD	Transylvania, Lower Danube	Garašanin 1975, 75	BelM429	MA-196303
Brezovo Polje	Socketed axe	Hoard	2	2747	Travnik Museum	BiH	Carpathian Basin, western Balkans	König 2004, 194	TravM 18	MA-152330
Brezovo Polje	Socketed axe	Hoard	2	2750	Travnik Museum	BiH	Carpathian Basin, western Balkans	König 2004, 194	TravM 19	MA-152331
Brezovo Polje	Spearhead	Hoard	2	2754	Travnik Museum	BiH	Carpathian Basin, western Balkans	König 2004, 195	TravM 27	MA-152337
Drenov Do	Socketed axe	Hoard-	2	21001	National Museum Sarajevo	BiH	Carpathian Basin, western Balkans	König 2004, 196	SJLM 89	MA-186736
Drenov Do	Sickle, type 3	Hoard	2	20991	National Museum Sarajevo	BiH	Carpathian Basin, western and central Balkans	König 2004, 197	SJLM 91	MA-186737
Drenov Do	Sickle, type 2	Hoard	2	20989	National Museum Sarajevo	BiH	Carpathian Basin, western and central Balkans	König 2004, 197	SJLM 93	MA-196011
Drenov Do	Bow fibula, type Golinjevo	Hoard	2	21003	National Museum Sarajevo	BiH	Western Balkans	König 2004.,197	SJLM 96	MA-186738
Drenov Do	Armlet	Hoard	2	20987	National Museum Sarajevo	BiH	Carpathian Basin, central and western Balkans	König 2004, 197	SJLM 97	MA-196012
Vojskova	Flange hilted sword, type Celldömölk	Singular deposition	2	32596	National Museum Sarajevo	BiH	Carpathian Basin, western Balkans, Italy	Harding 1995, 57	SJLM 63	MA-186729
Leskovo	Socketed axe	Hoard	2	154	National Museum Požarevac	SRB	Transylvania, Lower Danube, central Balkans	Todorović 1975, 78	PozM 20	MA-196051
Leskovo	Flange hilted sword, type Reutlingen	Hoard	2	155	National Museum Požarevac	SRB	Central Europe, Carpathian Basin, Balkans	Todorović 1975, 78	PozM 21	MA-186756
Leskovo	Neckring	Hoard	2	144	National Museum Požarevac	SRB	Central Europe, Carpathian Basin, Balkans	Todorović 1975, 78	PozM 23	MA-186757
Vojilovo	Spearhead	Hoard	2	62	National Museum Požarevac	SRB	Western and Central Balkans	Vasić 2015, 32	PozM 27	MA-186759
Sivec	Flange hilted sword	Grave	2	24	National Museum Skopje	MKD	Norther Aegean, Albania	Harding 1885, 54	SkoM 227	MA-186790
Demir Kapija	Bow fibula	Grave	2	43	National Museum Skopje	MKD	Adriatic area, North Macedonia, Albania	Vasić 1999, 45	SkoM 248	MA-196201
Gradac Sokolac	Bow fibula, type Golinjevo	Grave	3	97	National Museum Sarajevo	BiH	Western Balkans	Čović 1975, 34	SJLM 88	MA-186735
Grapska	Socketed axe	Hoard	3	1734	Doboj Museum	BiH	Carpathian Basin	König 2004, 198	DobM 46	MA-152347
Grapska	Razor	Hoard	3	1747	Doboj Museum	BiH	Western Balkans	König 2004, 198	DobM 48	MA-152349
Lučica	Decorated bracelet	Hoard	3	16674	National Museum Sarajevo	BiH	Western Balkans	König 2004, 207	SJLM 99	MA-196013
Ivanjska	Bow fibula, type Golinjevo	Singular deposition	3	3251	National Museum Sarajevo	BiH	Western Balkans	Čović 1975, 31	SJLM 25	MA-186708
Klaonica, Travnik	Bow fibula, type Golinjevo	Grave	3	3	Travnik Museum	BiH	Western Balkans	Gavranović & Sejfuli 2015, 72	TravM 32	MA-152339
Klaonica, Travnik	Neckring	Grave	3	7	Travnik Museum	BiH	Western Balkans	Gavranović & Sejfuli 2015, 73	TravM 36	MA-152341
Ilijaš	Sword, type Tešanj	Grave	3	-	Private collection	BiH	Western Balkans	Gavranović & Sejfuli 2018, 92	Pc 100	MA-195976
Pakline	Sword, type Veliki Mošunj	Singular deposition	3	30758	National Museum Sarajevo	BiH	Western Balkans	Harding 1995, 88	SJLM 61	MA-186727
Prozor	Socketed axe	Hoard	3	4	National Museum Sarajevo	BiH	Western Balkans	Žeravica 1993, 86	SJLM 23	MA-195990
Travnik	Bow fibula, type Golinjevo III, fragment	Grave	3	21	Travnik Museum	BiH	Western Balkans	Gavranović & Sejfuli 2015, 77	TravM 31	MA-152338
Veliki Mošunj	Anklet, repaired	Hoard-	3	31226	National Museum Sarajevo	BiH	Western Balkans	König 2004, 226	SJLM 65	MA-186730
Vitina, Otok	Bow fibula, type Golinjevo	Hoard	3	21836	National Museum Sarajevo	BiH	Western Balkans	König 2004, 216	SJLM 100	MA-196014
Vitina, Otok	Neckring	Hoard	3	21833	National Museum Sarajevo	BiH	Western Balkans	König 2004, 216	SJLM 102	MA-196015
Milci	Pin with bowl-shaped head	Grave	3	763	National Museum Skopje	MKD	North Italy	Vasić 2003, 94	SkoM 259	MA-196206
Novigrad	Plano-convex ingot	Hoard	4	32793	National Museum Sarajevo	BiH	Central Europe, Carpathian Basin, Balkans	König 2004, 216	SJLM 39	MA-195997
Novigrad	Plano-convex ingot with picking traces	Hoard	4	32791	National Museum Sarajevo	BiH	Central Europe, Carpathian Basin, Balkans	König 2004, 216	SJLM 48	MA-186719
Hetin	Plano-convex ingot	Hoard	4	3294	Vojvodina Museum Novi Sad	SRB	Central Europe, Carpathian Basin, Balkans	Koledin 2001, 37	VoNSM 221	MA-196190
Rudnik	Plano-convex ingot	Hoard	4	3275	National Museum Belgrade	SRB	Central Europe, Carpathian Basin, Balkans	Garašanin 1954, 31	BelM 449	MA-196318
Kličevac-Rastovača	Rod ingot	Hoard	4	2222	National Museum Požarevac	SRB	Carpathian Basin	Jacanović 2001, 39	PozM 35	MA-186761
Topolovaca Bregovi	Plano-convex ingot	Settlement	4	39	Doboj Museum	BiH	Central Europe, Carpathian Basin, Balkans	Belić 2010, 227	DobM 39	MA-152364
Podzvizd	Plano-convex ingot	Hoard	4	550	National Museum Sarajevo	BiH	Central Europe, Carpathian Basin, Balkans	König 2004, 216	SJLM 71	MA-196005
Podzvizd	Ingot fragment	Hoard	4	550a	National Museum Sarajevo	BiH	Central Europe, Carpathian Basin, Balkans	König 2004, 216	SJLM77	MA-196007
Rudnik	Plano-convex ingot	Hoard	4	3275b	National Museum Belgrade	SRB	Central Europe, Carpathian Basin, Balkans	Garašanin 1954, 31	BelM 447	MA-196317
Futog	Plano-convex ingot	Hoard	4	3419	Vojvodina Museum Novi Sad	SRB	Central Europe, Carpathian Basin, Balkans	Borić 1997, 65	VoNSM 187	MA-196167
Trlić	Plano-convex ingot, upper layer	Hoard	4	14465f_1	National Museum Belgrade	SRB	Central Europe, Carpathian Basin, Balkans	Garašanin 1954, 37	BelM 474	MA-196325
Trlić	Plano-convex ingot, lower layer	Hoard	4	14465f_2	National Museum Belgrade	SRB	Central Europe, Carpathian Basin, Balkans	Garašanin 1954, 37	BelM 475	MA-196326
Trlić	Rod ingot	Hoard	4	14464d	National Museum Belgrade	SRB	Carpathian Basin	Garašanin 1954, 37	BelM 467	MA-196323

Group field describes the number of the sampling group. Groups 1–3 correspond to the chronology of the objects. Group 4 includes the analyzed ingots. VIAS lab. no. is the identifier of the sample taken for this study. BiH = Bosnia-Herzegovina; SRB = Serbia; MKD = North Macedonia. CEZA Mannheim lab. no. is the identifier issued by the CEZA Laboratory in Mannheim. All necessary permits were obtained for the described study, which complied with all relevant regulations. Issuing authorities are listed in the field Museum/Depository.

### Group 1 (BA D–Ha A1)

**Fig 2:**
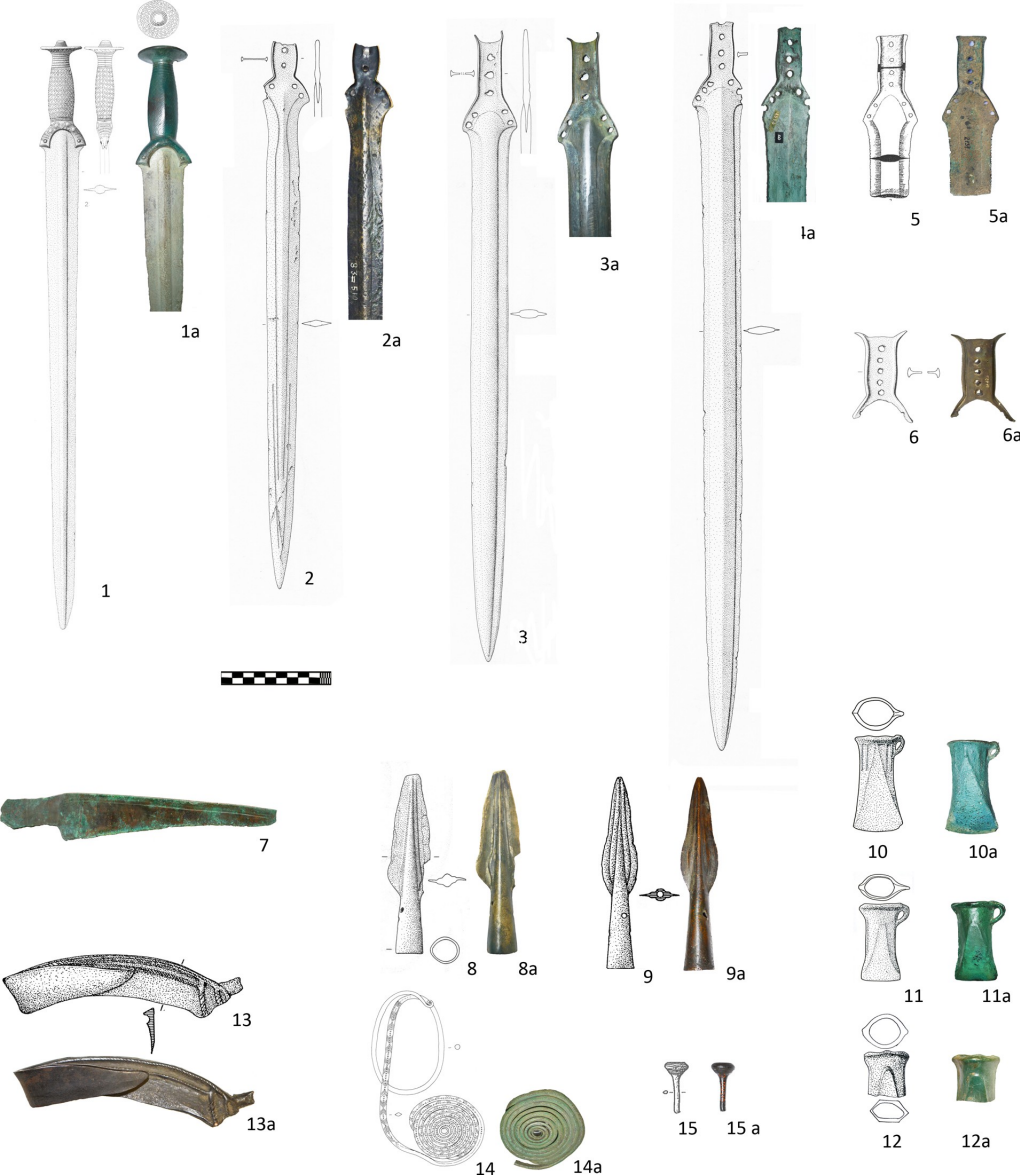
Group 1 of sampled metal objects (BA D–Ha A1). 1. 4. 10. 14: Topolnica; 2: Radaljska Ada; 3: Antonići; 5: Kličevac-Rastovača; 6: Trlić; 7: Mali Dol; 8: Novigrad; 9. 13. 15: Futog; 11. Urovica: 11; 21. Privina Glava (1–4. 6: reprinted from [41] under CC BY license with permission from A. Jockenhövel, original copyright (1995); 8: reprinted from [2] under CC BY license with permission from A. Jockenhövel, original copyright (2004); 9.13.15 reprinted from [65] under CC BY license, with permission of Vojvodina Museum Novi Sad, original copyright (1997); all other drawings and photos by authors M. Gavranović, D. Jacanović and A. Kapuran by permission of the Museum of Krajina Negotin (1a, 4a, 10-10a, 11-11a; 14-14a), National Museum of Bosnia and Herzegovina in Sarajevo (2a, 3a, 8a); National Museum in Požarevac (5-5a) National Museum in Belgrade (6a, 12-12a) Archaeological Museum of North Macedonia in Skopje (7) and Museum of Vojvodina in Novi Sad (9a, 13a, 15a).

The 15 objects in Group 1 depict the characteristic repertoire of metal finds in the study area between the 14^th^ and 12^th^ centuries BC with the prevalence of archaeological types distributed across the Carpathian Basin and central Europe and indicative of the Urnfield phenomena [14, 38, 40, 41, 46]. These include flange hilted swords of Aranyos and Reutlingen type (Fig 2: 2–6), two spearheads with characteristic leaf shape (Fig 2: 8–9), a sickle of Uiora 1 type (Fig 2: 13) and a pin with ribbed head (Fig 2: 15) [38, 40, 41, 52]. Thanks to the thorough typo-chronological studies, it is also possible to identify objects with specific regional traits (decoration, technical details) that suggest their production in local workshops. The swords from Kličevac-Rastovača with ricasso on the blade (Fig 2: 5) and from Trlić with an unusually high number of rivets (Fig 2: 6) are good examples of such local adaptations [41] [53]. The sword of Aranyos type from the Drina River (Fig 2: 2) is also most probably a local product with nearest analogies in Bosnia, Croatia and Serbia [41, 54].

The solid hilted sword of Riegese type and a spiral armlet from the Topolnica hoard (Fig 2: 1.14) are both atypical for the Balkans and are mainly distributed between the Alpine region and the Carpathian Basin [41, 55–59]. The possibility that these two items are products domestic workshops in thus not very high.

The three socketed axes with triangular and parabolic shaped ornament (Fig 2: 10–12) represent variations of a particular type that emerged in Transylvania and in the eastern Balkans between the Lower Danube and the Black Sea during the Ba D period [36, 59–63]. The analyzed axes from the Serbian sites of Topolnica, Urovica and Privina Glava mark the western frontier of the general distribution and are selected as representatives of a techno-typological metallurgical circle that clearly point toward the regions situated in the East of the study area.

Finally, in order to get insight in the metallurgical circles of this time span in North Macedonia, we also included a knife from Mali Dol in the Group 1 as a strictly local type, found mostly at the sites along the middle Vardar valley and thus probably also produced in the same region [64].

### Group 2 (Ha A2–Ha B1)

The chosen 15 samples forming Group 2 reflect the range of the metal finds of the 11^th^ and 10^th^ centuries BC from the study area in terms of distribution tendencies, provenance, typology and function.

**Fig 3:**
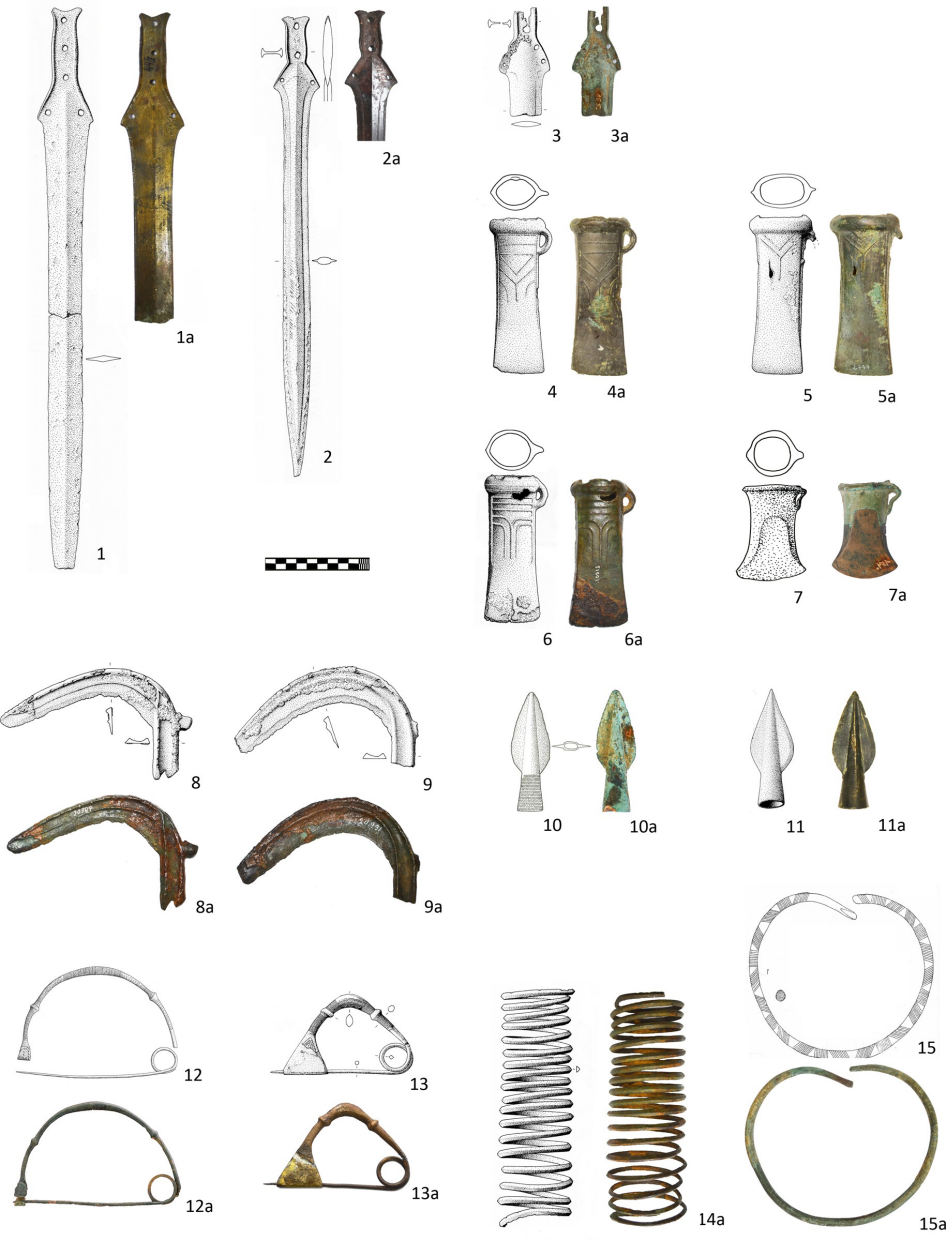
Group 2 of sampled metal objects (Ha A2–Ha B1). 1. Sivec; 2: Vojskova; 3. 7. 15: Leskovo; 4–5. 11: Brezovo Polje; 6.8–9.13–14: Drenov Do; 10. Vojilovo; 12: Demir Kapija (1–3: reprinted from [41] under CC BY license with permission from A. Jockenhövel, original copyright (1995); 4–6. 8–9. 11. 13–14 reprinted from [2] under CC BY license with permission from A. Jockenhövel, original copyright (2004); 12: reprinted from [70] under CC BY license with permission from A. Jockenhövel, original copyright (1999); 15: reprinted from [71] under CC BY license with permission from A. Jockenhövel, original copyright (2010): all other drawings and photos from authors M. Gavranović and A. Kapuran with permission of Archaeological Museum of North Macedonia in Skopje (1a); National Museum of Bosnia and Herzegovina in Sarajevo (2a, 4a–6a, 8a-9a, 11a, 14a); National Museum in Požarevac (3a, 7-7a, 10a. 15a) Archaeological Museum of North Macedonia in Skopje (12a).

Representative for metal types of wide distribution between central Europe, the Carpathian Basin and the Balkans are two flange hilted swords from Vojskova and Leskovo (Fig 3: 2–3), socketed axes with Y-shaped ribs from Brezovo Polje and Drenov Do (Fig 3: 4–6), two sickles from Drenov Do (Fig 3: 8–9) and two short spearheads from Vojilovo and Brezovo Polje (Fig 3: 10–11) [2, 36, 40, 41, 52, 66]. Although embedded in the supra-regional typological cannon and closely connected with the networks of the younger Urnfield period, most of these objects are most probably products of local workshops. This is particularly indicated by the casting moulds from the domestic workshops in Varvara or Lovas [3, 36] and by some technological and decorative details (e. g. decoration of the spearhead, Fig 3: 10) that are characteristic for the metal items spread between the Sava and Danube Rivers and Adriatic Sea [40].

On the other hand, the sword form Sivec (Fig 3: 1) points at the specific regional production and distribution network that includes the territories of North Macedonia, northern Greece and Albania [41, 67]. There is a little doubt that such swords are a part of the local metallurgical tradition of this area that also continued to exist in the following Early Iron Age [67].

The socketed axe from Leskovo (Fig 3: 7) represents a continuing influence of the metallurgical circle from the regions of Transylvania and the Lower Danube [36, 68, 69]. However, finds of casting moulds for corresponding axes in northern Serbia indicate that from Ha A2 stage onwards local workshops were also engaged in the production of diverse variations of this type [36], pointing at the possibility that the sampled axe was casted not too far away from the place of deposition.

A one-loop bow fibula from the grave in Demir Kapija is a specific type with a distribution between Italy, the Adriatic coast, Albania and northern Greece [49, 50, 70]. The sampled fibula belongs to a so-called “Macedonian series” found in graves along the Vardar valley. Although this might suggest local production, fibulae in graves often result from female mobility, meaning that this particular piece could also originate from the Adriatic area or from Italy. Conversely, the second bow fibula from Group 2 (Fig 3: 13) is a strictly local jewelry type with a relatively narrow distribution in the territories of central and eastern Bosnia and is thus very likely a local product [17, 18]. The arm spiral from Drenov Do. (Fig 3: 14) and the decorated neck ring from Leskovo (Fig 3: 15) are both characteristic for local female grave sets and are with high probability also made by local foundries [2, 69].

### Group 3 (Ha B3)

**Fig 4:**
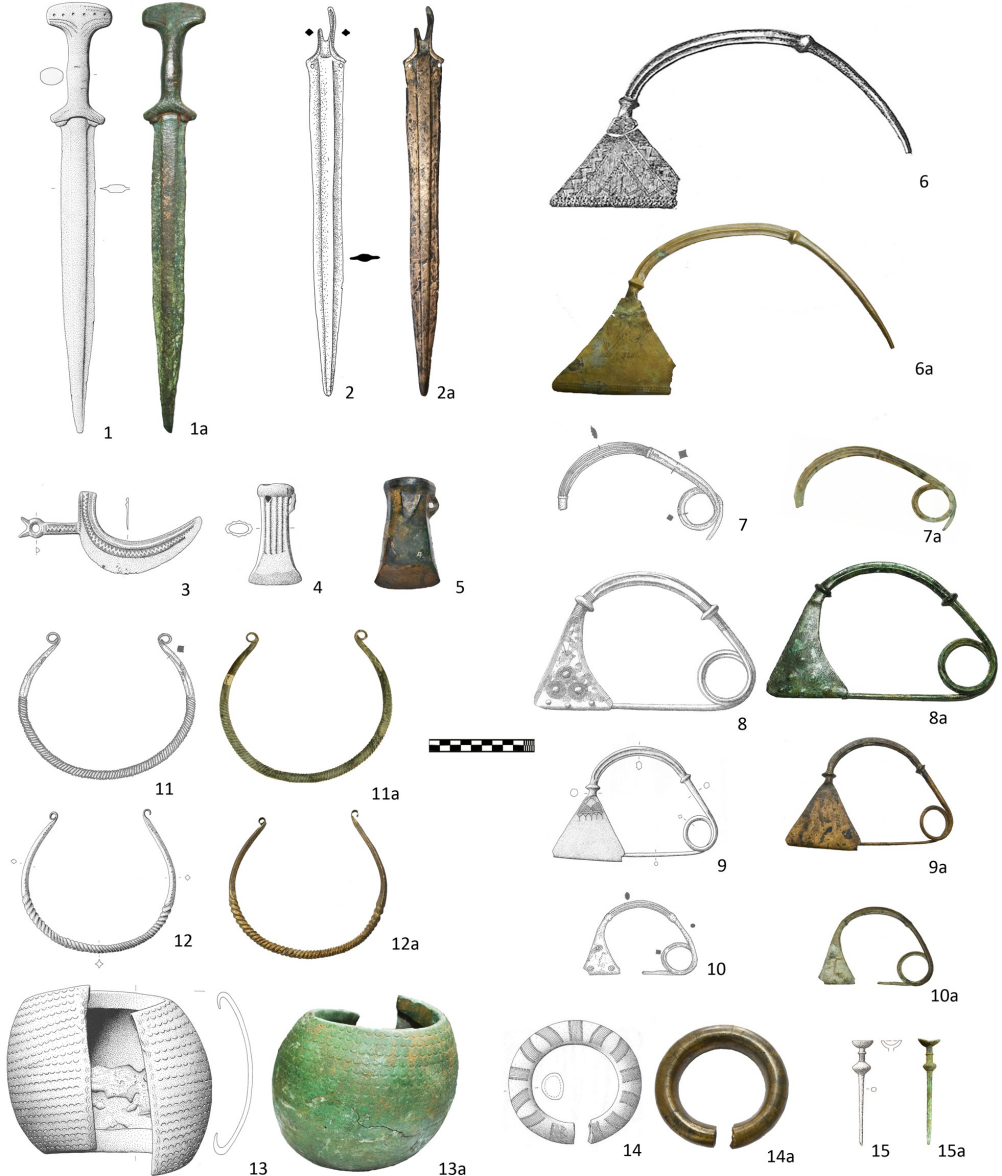
Group 3 of sampled metal object (Ha B3). 1. Pakline; 2. Ilijaš; 3–4: Grapska; 5. Prozor; 6. Ivanjska; 7. 10. 12: Travnik; 8: Gradac Sokolac; 9.12: Vitina-Otok; 13: Veliki Mošunj; 14: Hrge-Lučica; 15: Milci (1: reprinted from [41] under CC BY license with permission from A. Jockenhövel, original copyright (1995); 3–4. 9. 12–13: reprinted from [2] under CC BY license with permission from A. Jockenhövel, original copyright (1995); 15: reprinted from [38] under CC BY license with permission from A. Jockenhövel, original copyright (1995).

The third group of samples also comprises 15 artefacts that mirror the general trend of the 9^th^ century BC (Ha B3) with an almost exclusive occurrence of local bronze types. While connection to the neighboring areas of the Carpathian Basin or central Europe are less apparent in the spectrum of metal finds, some of the bronze types that are characteristic for the Apennine Peninsula started to appear across the western and central Balkans, pointing to intensification of exchange and mobility between the two shores of the Adriatic. A bronze pin with bowl-shaped head from Milci cemetery in the Vardar valley (Fig 4: 15) is of a type that is actually characteristic of North Italy and hence is evidence of increasing contacts with that region [38, 72].

As one of the few metal types of this period that has no real analogies in the Balkans, we also included one socketed axe from the Grapska hoard in the sample selection (Fig 4: 4) as an example of a metal object that could originate from some exchange network outside the study area [2].

The remaining 13 objects from the Group 3 are distinctive archaeological types with a primary distribution in the Dinaric Alps between the Sava River and the Adriatic. The sampled short swords (Fig 4: 1–2), razor (Fig 4: 3), socketed axe (Fig 4: 5), bow fibulae (Fig 4: 6–10), arm rings and torcs (Fig 4: 11–14) are all evidence of thriving local metallurgy that yielded a variety of new types [2, 11, 18, 73]. Many of these objects are lavishly decorated and/or made in oversized proportions (e.g. a fibula from Ivanjska measuring 30 cm or a massive bracelet from Veliki Mošunj weighing nearly 2kg), which underscores the prestigious character and higher social rank of their holders. In terms of decoration and shape, some of the selected items from Group 3, such as the razor from Grapska (Fig 4: 3) or the short swords from Pakline and Ilijaš (Fig 4: 1–2), are distantly related to the contemporary finds from Italy, but are all distinct regional archaeological types undoubtedly cast and decorated in the local workshops [18, 73]. An additional source of evidence are the corresponding casting moulds from the sites situated in the distribution area of the artefacts [3].

### Group 4 (copper ingots)

Within our dataset, we were able to analyze ingots from the hoards of the Ba D–Ha A1 period and a few contemporary pieces from the settlements, while younger finds (Ha B1 or Ha B3) are still lacking. Considering that copper ingots, and more often their fragments, occur in abundance during stages of the Late Bronze Age across the Carpathian Basin, the limited number of finds from periods after Ha A1 in the western and central Balkans is striking [74]. The sample set presented here includes nine plano-convex ingot fragments as the most frequent shape, two rod ingots and one ingot fragment. The plano-convex ingots were selected because they either originate from a workshop area in the settlement such is the find from Topolovaca Bregovi [75], or they have cutting marks like a fragment from the Novigrad hoard [2]. Furthermore, we also analyzed ingots from Rudnik, Trlić, Futog and Podzvizd that are interesting in terms of their deposition on the most southern fringe of the general hoard distribution of this period [14, 76]. The ingot fragment from the Hetin hoard appears intriguing because this deposit contains weapons, jewelry and tools not typical for the Balkans but rather for the eastern part of the Carpathian Basin [77]. Finally, the rod ingots from Trlić and Kličevac-Rastovača stand out as few pieces of this kind have been found south of the Save and Danube Rivers, while such ingots occur in much larger numbers across the Carpathian Basin [53, 78].

## Methods

The metal samples were taken with a polished 1 mm stainless steel drill bit. Before use, each was cleaned with highly concentrated ethyl alcohol (97%) to remove possible remains from the production process (e.g. polishing emulsion). As a first step, the patina was removed with a drill and afterwards a new bit was used to drill a 1–2 mm deep hole. Finally, the resulting drill-shavings were collected. If corroded material was still present in the sample, it was removed under a microscope.

All samples were analysed at the Curt-Engelhorn-Zentrum Archäometrie (CEZA) in Mannheim. For the measurements of the trace element concentrations an energy dispersive X-ray fluorescence (EDXRF) spectrometer (ARL Quant X, Thermo Scientific) was used to analyse the elements silver (Ag), arsenic (As), bismuth (Bi), cobalt (Co), copper (Cu), iron (Fe), manganese (Mn), nickel (Ni), lead (Pb), selenium (Se), tin (Sn), antimony (Sb), tellurium (Te) and zinc (Zn). The following reference materials of the BAM–Bundesanstalt für Materialforschung und–prüfung (BAM-367, BAM-368, ERM-EB374, ERM-EB375, BAM-376) and various in-house standards were used for calibration. Each sample was measured in two exposures of 600 seconds; two standard materials (BAM 211 and BAM 376) were included in each run. The measurements were normalised to 100%. The detection limits lie at about 0.005% for Ag, Sb, Sn, Pb, Bi and 0.05% for Fe as well as around 0.01% for Co, Ni, and As. Se, and Te were measured, but were below 0.005% in all samples. Zn was below the detection limit of 0.1% in all samples, which was due the spectral interference with copper. Sulfur was not measured [28].

The lead isotope ratios (^208^Pb/^206^Pb, ^207^Pb/^206^Pb and ^206^Pb/^204^Pb) were measured by using a high resolution multi-collector mass spectrometer (Thermo Scientific Neptune Plus) with inductively coupled plasma as ion source (HR-MC-ICP-MS). The ^208^Pb/^204^Pb, ^207^Pb/^204^Pb ratios were calculated from the afore mentioned measured ratios. The drill shavings were rinsed with dilute HNO_3_ to remove any surface contamination, then dissolved in half-concentrated HNO_3_ in an ultrasonic bath (70˚C) for several hours. Insoluble residues were removed by decantation, afterwards the solution was diluted with deionised water. Ion exchange columns were prepared with PRE-filter and Sr-resin and were preconditioned with 500 μl 3N HNO_3_ before the solution was added. In four steps, the matrix was first eluted using HNO_3_, and then the Pb was eluted using HCl. The mass fractionation of lead is corrected by the addition of thallium (Tl), for which a ratio of ^205^Tl/^203^Tl = 2.3871 and an exponential fractionation behaviour are assumed. The interference of ^204^Pb and ^204^Hg was corrected by measuring ^202^Hg with a ratio of ^204^Hg/^202^Hg = 0.2293. The in-run precision of the measurement was typically 0.02 to 0.05% (2σ), depending on the isotopic ratio. The reference sample NIST SRM 981 was measured after each eight samples to check for instrumental drift and guarantee high-level precision and accuracy [28]. The methods are described in detail by Lutz and Pernicka [79] and Niederschlag et al. [80].

The elements Ni, As, Sb, Ni and Bi were combined to double logarithmic diagrams, the ^206^Pb/^204^Pb, ^207^Pb/^204^Pb and ^208^Pb/^204^Pb lead isotope ratios were used to generate bivariate comparative lead isotope plots for the discussion of the data. The tin and lead concentrations served as parameters for the description of the alloying practices. First, the archaeological information (context, chronology, typology, spatial distribution) was combined with the chemical and isotopic data of the artefacts under study in order to define groupings and dispersion. Afterwards the possible provenance of the copper-based objects was examined by comparing their geochemical information with those of known ore deposits in the regions under study. The final theory based archaeometallurgical and archaeological interpretation focused on the discussion of the proposed provenance of the copper and its implications concerning alloying, recycling, metal circulation and exchange in a supra-regional perspective [28, 81].

## Results and discussion

### Lead isotope analyses, provenance studies and exchange networks

In our previous studies, we demonstrated that during the Early and Middle Bronze Age (1^st^ half of the 2^nd^ millennium BC) copper was produced in the region of eastern Serbia near the city of Bor, where abundant ore deposits have been exploited until the present day [12, 13]. We also provided evidence that between 1900 and 1600 BC the metal from eastern Serbia was a part of the regional exchange network as several analyzed bronze objects from the Balkans were clearly made of the copper form these sources [13]. At around 1600 BC, the copper production in eastern Serbia ceased and metal with a different geochemical signature entered the western and central Balkans. Our previous work revealed that metal objects made from copper smelted in the regions of the southern Alps (Trentino, South Tyrol) and in the eastern Alps (Hochkönig–Mitterberg) started to appear in the Balkans [13].

Following the work of G. Artioli [82] we use the term “southern Alps” for the ore districts in Trentino, southern Tyrol (Alto Adige) and Veneto (AATV and Valsugana VMS), while “eastern Alps” refers to regions in Austria (especially the Hochkönig-Mitterberg area).

**Fig 5:**
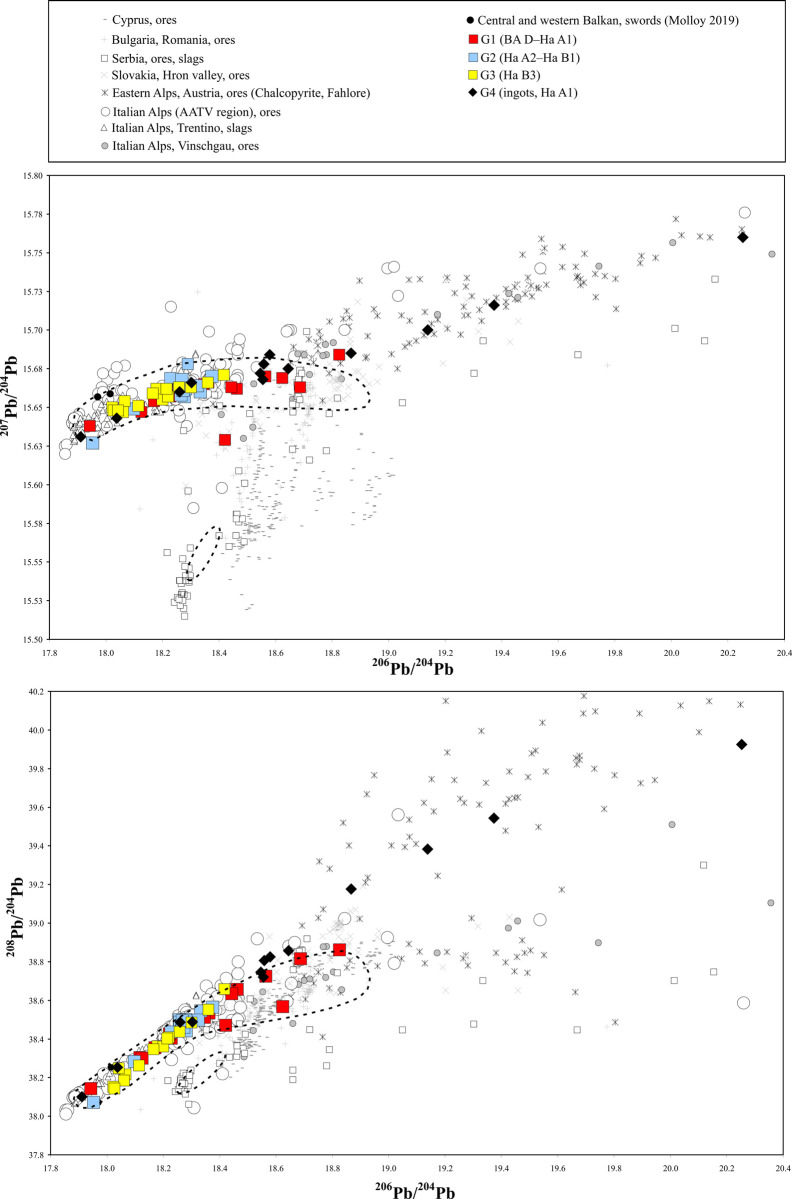
LI diagrams comparing LI signatures of the central and western Balkan objects under study with other major European, Balkan and Mediterranean ore fields. In the lead isotope diagrams, the artefact data from Groups 1–4 (58 squares and diamonds) is compared with those of ores and slags found in the Alpine region as well as in the Balkan area. The dotted ellipse circumscribe the lead isotope ratios of 134 additional LBA objects within the dataset. The three above mentioned objects that point to the copper ores from Cyprus are excluded from ellipse [89, 90] It becomes obvious that the vast majority of the LBA objects under study is consistent with the ore deposits in the southern and eastern Alps. The black diamonds represent the ingots found in Bosnia and Herzegovina and Serbia, discussed in this study (Table 1). The ore and artefact data are from [9, 23, 82–84, 93, 94]. The analytical uncertainty is equivalent to or smaller than the size of the symbols (chart: M. Mehofer, University Vienna).

The extent and intensity of this new metal input in the exchange systems of the Late Bronze Age could only be speculated and therefore further systematic examination was required. The research presented here makes it possible to elucidate analytically based insights into metal exchange networks of the regions under study. Furthermore, it provides a starting point for reconstructing the dynamics of the copper supply networks in the research area during the second half of the 2^nd^ millennium BC. In the following, the trace element and lead isotope analyses of the LBA artefacts and ingots discussed in this study (S1 and S2 Tables) are compared with those of ore deposits situated on the Balkan peninsula, on Cyprus and in the Alps [19, 23, 82–87]. These deposits are most likely the sources for the copper used in the study region. The dominant presence of copper from the southern Alps is–as demonstrated by Fig 5 –attested by analytical means. The vast majority of our artefacts fall in the same isotopic range of copper smelting slags and ores from the Valsugana volcanogenic massive sulphides (VMS) and the AATV district (Alto Adige, Trentino, Veneto) (Fig 5) [20, 82, 86].

This is also true for a few objects published in other studies such as a sword from Hajdukovo in Serbia and another from Sisak in Croatia [9]. The provenance of their copper can also be narrowed to the ore deposits of the Valsugana VMS district, Trentino (Fig 5). Unfortunately, only pXRF and SEM-EDS were used to determine the trace element concentrations, which is why the data is not fully comparable to the ED-XRF results used in this present study.

As will be described below, our work has also found that the ore fields in Bulgaria and Romania can be excluded as a significant supplier of raw material [84, 88]. Within our sample set, only a minor number of objects have geochemical characteristics that point to these copper sources. Exceptionally, some of the objects examined contained copper from Cyprus, such as two ingots from the Kličevac-Rastovača hoard in Serbia and the Paležnica hoard in Bosnia, as well as one Mycenaean rapier from Tetovo in North Macedonia [89, 90].

**Fig 6:**
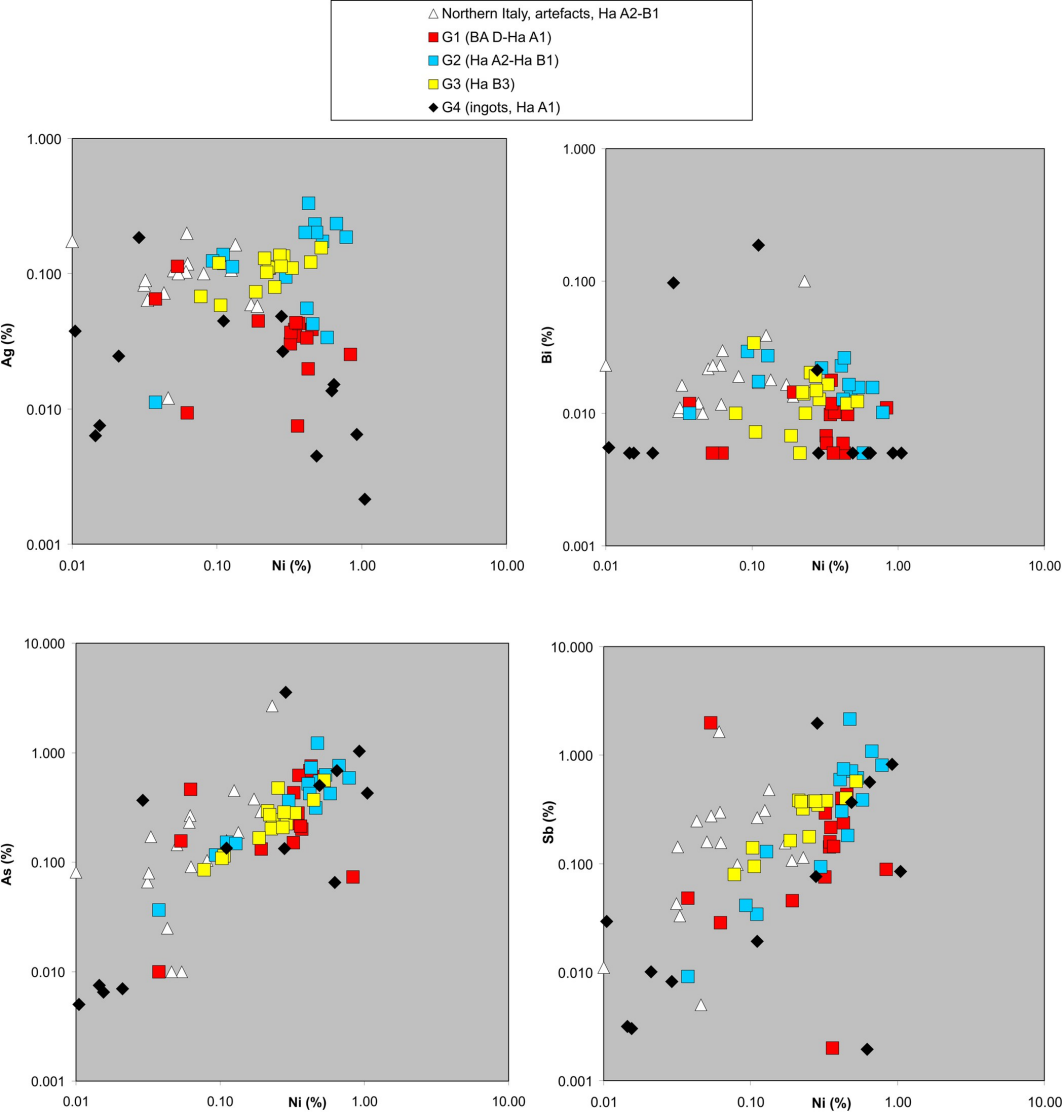
Trace element values of the artefacts under study compared to those of Northern Italian objects; The double logarithmic diagrams show the analytical results of the finds under study (squares), compared with those of objects which were found in Northern Italy (white triangles). The artefact data is from [20]. All values are given in weight % (chart: M. Mehofer, University Vienna).

The investigated ingots from Group 4 present a more nuanced picture (S1 and S2 Tables). It is generally accepted that the ingots were produced at or near the copper smelting sites and thence traded to the areas outside their production region [91]. The distribution of the ingots shows the high density in or around mining areas. The primary produced copper was melted and within one or more tapping/casting events casted into a bowl-shaped mould [92]. The plano-convex ingot from Trlić (VIAS lab. no. BelM 474 and 475) can serve as an example for this practice. In the cross section of this ingot, two layers resulting from two casts are visible. The XRF and lead isotope analyses from both layers revealed almost identical chemical composition, indicating that the metal comes from the same charge or source. Occasionally, scrap metal was also added during the secondary casting process [92]. In our case, this is to assume for the ingot from Topolovaca Bregovi (VIAS lab. no. DobM 39) with ca. 1% tin. Nevertheless, the geochemical composition of the ingots can generally be seen as indicative of the copper raw material that was fed into the exchange networks. Regarding our data it is to observe some of the ingots, including the finds from Novigrad, Hetin and Rudnik hoards (VIAS lab no. SJLM 39, SJLM 48, NovSM 221 and BelM 449), have lead isotope ratios comparable to the eastern Alpine mining areas (e.g. Hochkönig-Mitterberg), whilst ingots found in Kličevac-Rastovača, Topolovaca Bregovi, Podzvizd and Rudnik (VIAS lab. no. PozM 35, DobM 39, SJLM 71, BelM 447), are consistent with the isotopic data of the mines or slags found in Trentino [13]. This separation can also be followed in the trace element diagrams (Fig 6).

The remaining analyzed ingots (VIAS lab. no. SJLM 77, VoNSM 187, BelM 467, 474 and 475) have ^206^Pb/^204^Pb ratios between 18.464–18.658, and ^207^Pb/^204^Pb values in the range of 15.659–15.683, which places them in the area where ores from the southern and eastern Alps overlap, but the data also loosely points to ores from the Hron valley (Slovakia), Bulgaria, Romania and Serbia (Fig 5). Additionally, few artefacts also have lead isotope values within this range, all of them from Group 1 and thus dating between 13^th^ and 12^th^ century BC (Fig 5). On the one hand, one can hypothesize that the metal of these finds derives from Alpine ore deposits as e.g. ores from the Vinschgau region lie within this isotopic section. On the other hand, we have to point out that the lead isotope data of these objects could also be associated with e.g. deposits in the Hron valley in Slovakia [93] or with deposits in Serbia [23]. Therefore, at the current state of research, these areas should also be considered as possible source regions for some of the metals from Group 1.

Another explanation as to why the data points of some pieces are situated in the afore mentioned isotopic range might be that some of these objects are the product of mixing or recycling of copper of different provenances e.g. from the southern Alps with copper of Bulgarian, Serbian or eastern Alpine origin. However, this can widely be excluded for the ingots with lead isotope ratios situated in the range above as they represent–as generally described—raw material produced near of the copper smelting sites. It is therefore unlikely that they are the result of mixing of raw materials originating from regions far apart from each other (e.g. the Trentino and the Hochkönig-Mitterberg area). This does not exclude the possibility that the copper from two closely situated smelting sites was melted together. The subsequent processing of the metal can of course alter its chemical composition and result in the presence of additional elements such is e.g. tin. The analyzed ingots VIAS lab. no. BelM 467 and PozM 35 contain such additional alloying agents that came from melting events during which e. g. bronze was added, recycled or remained in the crucible and eventually mixed with the copper.

Another possible interpretation for the ingots situated in the overlap zone between the southern and eastern Alps and the Balkans is that they could derive from some ore district that has still not been fully characterized from an archaeometallurgical point of view. As an example, the recent research on ore deposits in the southern Alps demonstrated that further archaeological and archaeometric investigation on possible prehistoric mining regions may still provide new insights and data. In their well-conceptualized study, G. Artioli and his co-authors examined ore deposits in the Carnian, the Trentino and the Veneto as well as in the Vinschgau in Southern Tyrol [82]. The latter ore fields are underrepresented in the dataset with only six samples, which is not enough to get a conclusive picture on the lead isotope variations of a specific ore district. As some of them contain radiogenic lead, they additionally have a wide isotopic variation. First the recently published research by Th. Koch Waldner and M. Mehofer enlarged the available dataset and allowed a comprehensive picture of the ore deposits and their mineralization in the AATV region to be built [94].

Regarding the ingots that correlate with eastern Alpine copper, it is remarkable that almost none of the finished metal objects has lead isotope ratios that fully match with this area. A closer look at the trace element composition and especially at the lead concentrations of the ingots might help to explain this. Our dataset shows that the ingots that are fully comparable with the AATV and Valsugana VMS mining areas in the Trentino, have lead concentrations (mean 0.052%, median: 0.046%, n = 4) that are generally higher than those of the eastern Alpine ingots associated with the Hochkönig-Mitterberg region (mean 0.023%, median 0.005%, n = 4). However, if the ingots from both regions were mixed in the course of production, the isotope values of the ingots assigned to the southern Alps would still dominate due to their higher lead concentration such that eastern Alpine isotope signature would be erased. This might be one of several explanations as to why nearly no finished metal objects can be assigned to the deposits in the eastern Alps.

### Trace element analyses, copper-types and alloying practices

The analytical examination provides further insights into the distribution of metal in the western and central Balkans, and points to certain changes that occurred within the considered time span. As a first step, bismuth, arsenic, silver, antimony and nickel were combined with the specific double logarithmic trace element diagrams with the aim of verifying if certain groups can be described. The element pattern from our dataset indicates a chalcopyrite ore basis. Typical fahlore signatures with elevated Sb and As values appear rarely. In general, it is to state that fahlore-based metal objects are a minority in our dataset.

The 45 objects from Groups 1, 2 and 3 also serve as a case study in order to examine how closely they follow the observed general analytical trends during the European Bronze Age, if at all (Figs 5 and 6). Considering the lead isotope values of the artefacts, it is obvious that most of them correspond with ores from the southern Alpine mining districts, regardless of their date, function or distribution (Fig 5). Some of the analyzed ingots and finished objects reveal a diverse trace element pattern with element concentrations different from their group´s average, opening the possibility that the metal from some other copper sources was used in their production. The lead isotope analyses indicate that six finds s from Group 1 contain copper of a different provenance than most of the objects. These are: a sword of Riegesee type (VIAS lab. no. NegM 6), a spiral armlet (VIAS lab. no. NegM 17) and a socketed axe of Transylvanian type (VIAS lab. no. NegM 99) all coming from the Topolnica hoard, as well as a socketed axe from Urovica (VIAS lab. no. NegM 24), a socketed axe found in Privina Glava (VIAS lab. no. BelM 429), and a knife from Mali Dol (VIAS lab. no. SkoM 276). Given the differing lead isotope ratios it is less surprising that the chemical composition of these six artifacts also varies in relation to other finds (Figs 5 and 6).

The most distinct outlier is the knife from Mali Dol in North Macedonia (Fig 2: 7), which displays some consistency with ores from the eastern Alps but has no clear match, and is marked by a lower concentration of nickel and silver. Since this is a local form typical for sites along the Vardar valley, one has to evaluate if the copper used in the production could originate from some nearby source that has still not been documented in terms of lead isotope analyses. Another outlier that is clearly separated from the cluster of other objects and isolated in relationship to all of the copper ore sources thus far known is a sword of Riegsee type from Topolnica (Fig 2: 1). This sword is characterized by very low Ag and Sb values, whilst the armlet from the same depot not only shows somewhat different lead isotopic values than most of the analyzed objects, but also has Ag, As and Ni concentrations well below the average of the chronological group (Fig 6). As indicated in the typological and distributional summary above, the sword and the armlet are not a part of the regional repertoire and whilst the lead isotope and chemical data confirm its production outside of the Balkan metallurgical network, the metal provenance currently remains unclear.

The archaeometallurgical analyses of the socketed axe of Transylvanian type also from the Topolnica hoard (Fig 2: 10), separates this artefact from the others in Group 1. Its lead isotope ratios relate it with ores from Serbia and the elevated Ni concentration of 0.84% supports the conclusion of a differing metal provenance (Fig 6). The higher trace element concentrations–especially the Sb and Ag values–of a socketed axe from Urovica (Fig 2: 11) clearly confirm that this object is made of fahlore derived copper. The examination of the lead isotope ratios additionally separates it from the artefacts with the copper from the southern Alps but do not allow a direct association with any other known deposit. The socketed axe found in Privina Glava (Fig 2:12) also do not show any clear isotopic match, but its trace element pattern point to a chalcopyritic ore basis, which is also the case for most of the other objects from Group 1. Worth mentioning is the fact that objects with differing lead isotope ratios indicating some other copper source are all from Group 1 (BA D–Ha A1), while copper from the sources in the southern Alps clearly prevails in the metal artefacts from subsequent periods (Group 2 and 3) (Fig 5).

In Group 2, three finds from the Leskovo hoard in Serbia (VIAS lab. no. PozM 20, 21 and 23) do not have the same trace element values as their specific chronological group. In terms of silver values, they in fact correspond more closely to the cluster of Group 1.

This is not unexpected since all three objects (a flange hilted sword, a neck ring and a socketed axe) (Fig 3: 3.7.15) are typologically older (BA D–Ha A1). Due to their deposition with some younger finds, they were assigned to the next chronological phase (Ha A2–Ha B1). The occurrence of the older objects in the deposits, which are mostly assemblages of items collected throughout a certain time, is anything but unusual [14, 95]. As such, the finds from Leskovo serve as an excellent example for the need to integrate metallurgical analyses within archaeological research of such objects, Although unlikely in this instance, one must also acknowledge that such an analytical result could also be caused by re-melting of older objects during a subsequent chronological period.

The only object type from the remaining two groups 2 and 3 that does not match with the main cluster is the sword from Sivec (Fig 3: 1, VIAS lab. no. SkoM 227). Even though the lead isotope data would at least permit the assumptions that the copper originated from northern Italy, its trace element composition is not comparable to any of the finds with a southern alpine geochemical pattern. It has a significantly lower concentration of Ag, Sb, As and Ni (Fig 6). The distribution of similar swords in North Macedonia, Greece and Albania suggests a regional metallurgical network that apparently procured copper from another source compared to that used by the majority of workshops in the western and central Balkans.

### Copper-types: Low-impurity chalcopyrite-like copper and high impurity fahlore-type copper

Coming back to the trace element pattern of the objects examined in our study, one can state that in the As-Ni and Sb-Ni diagrams a nearly 1:1 correlation of these elements is recognizable. This applies for all samples regardless of their distribution or typology. There is no clear separation in terms of chronology. However, one can assume that the higher Sb and As concentrations are connected to the use of ores with accessory minerals containing these elements. For example, the main lode at the Hochkönig–Mitterberg mining areas (one of the source regions of the analyzed objects) is associated with three stages of ore formation, one of them being nickel-rich pyrite (FeS_2_). The second is chalcopyrite, which is the dominate ore type and the third are cobalt-rich copper ores [19]. In the southern sector, the ore mineralisations strata contain accessory minerals like gersdorffite (NiAsS), ullmanite (NiSbS), millerite (NiS) and arsenopyrite (FeAsS) and fahlore (tetrahedrite–tennantite) [19] [96, 97]. Nickel—containing fahlore is additionally known from the Inn valley [85, 96]. Ores with various accompanying minerals are registered also in the southern Alps, the major source region of the artefacts under study, e.g. from Montagiù or Miniera Bedovina [82]. Ullmannite, a Ni-Sb bearing mineral is also attested for the copper ores in the Schneeberg region in southern Tyrol [97]. During the smelting process, these elements incorporate into the metal in various amounts,–depending on the concentration of the accessory minerals [98].

The second possible explanation is that mixing or recycling of metal with different provenance (e.g. Ni-bearing fahlore and chalcopyrite copper) resulted in copper with this composition. If metals from the eastern Alpine mining areas (e.g. Hochkönig-Mitterberg) and from Trentino were mixed, as has been assumed for some of the artefacts, the concentrations would change and result in elevated or decreased element values. Their element values would be situated along a mixing line or section as visible in the Sb-Ni diagram (Fig 6). Admittedly, the number of ingots presented in this study is too small to draw such general conclusions.

**Fig 7:**
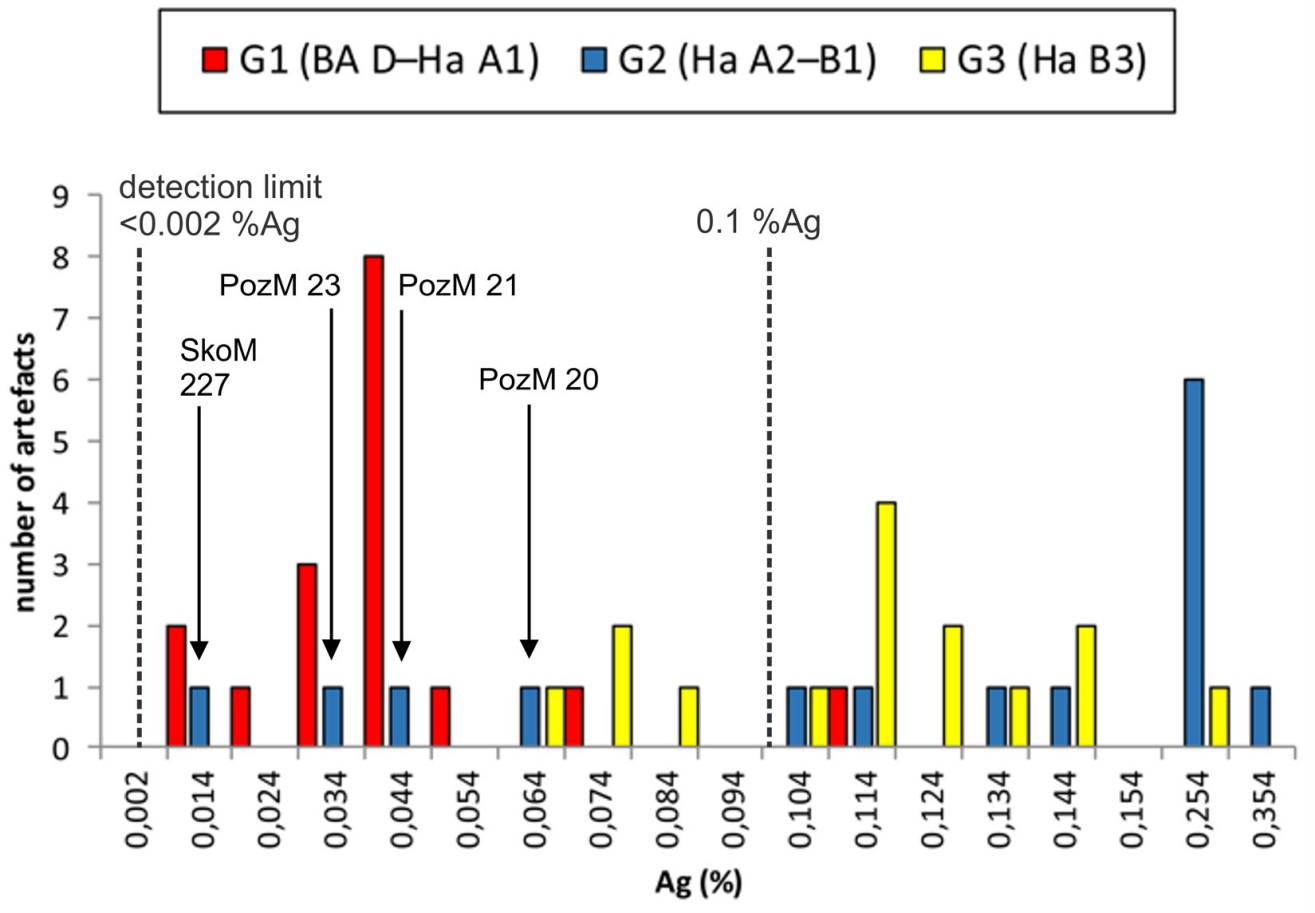
Silver concentration of the objects presented in this study, subdivided by chronology. Silver concentration in the 45 pieces under study. The two peaks at 0,044% and 0,254% represent two types of copper (n = 45). Note that the chronological group Ha A2–B1 (blue columns) contains four objects, which are dated to BA D–Ha A1 (red columns) by typology, but were found in younger contexts. They are geochemically consistent with pieces assigned to the older phase BA D–Ha A1. All values are given in weight % (chart: M. Mehofer, University Vienna).

In contrast, the silver-nickel diagram of our samples points to the existence of two distinct groups separated by their silver concentration (Fig 6). This chemical divergence coincides with specific chronological groups as outlined by the typological and distributional evaluation above (Fig 7). Generally speaking, artefacts dated to BA D–Ha A1 and earlier have Ag concentrations below 0.08–0.1 weight%, whilst the younger objects (Ha A2– Ha B1 and Ha B3) tend to have elevated silver concentrations up to 1 weight% (S1 Table).

The fact that objects from different chronological groups deviate when it comes to Ag and Sb concentrations but are more or less consistent in terms of lead isotope values can be explained in multiple ways.

**Fig 8:**
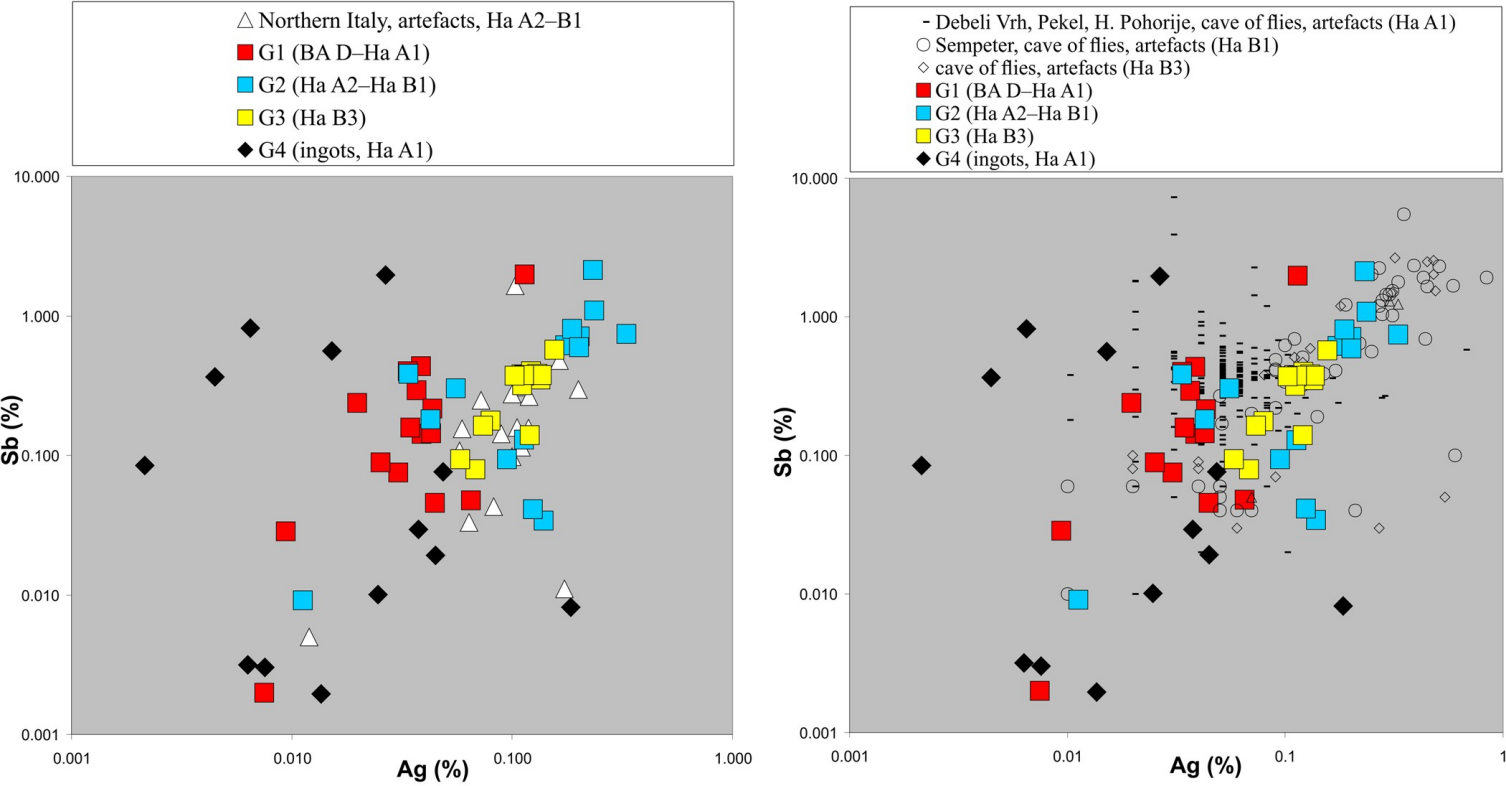
Comparison of the antimony-silver values of Northern Italian, Balkan and Slovenian artefacts. The left picture displays the antimony-silver concentrations found in objects from Northern Italy and the western and central Balkans subdivided by their chronology. The right presents the same combination with the Slovenian artefacts. A comparable trend–younger dated pieces have higher Sb and Ag is describable. All values are given in weight % (chart: M. Mehofer, University Vienna).

Firstly, this can be caused by a change in the exploited and smelted copper ore basis from chalcopyrite to fahlore-dominated ores. Secondly, instead of chalcopyrite, ores with additional minerals could have been mined and smelted, which would also result in elevated concentrations of specific trace elements. Most recently, a further explanation came back into discussion [96, 99, 100]. C. Grutsch and co-authors discussed the analytical results of Early Bronze Age to the Early Iron Age ingots and finished objects from the western Austria (Vorarlberg, Northern Tyrol, Salzburg, Upper Austria) [100]. While chalcopyrite copper with low impurities dominates in the MBA and early LBA artefacts, another trace element pattern appears from Ha A1/Ha A2 onwards. During the phases Ha A1–Ha D2 (1100–500 BC) copper with elevated silver and antimony concentrations prevails, yet the Ag and Sb are too high to derive from chalcopyrite, and too low to come from fahlore. Therefore, Grutsch and her co-authors argue that the mixing of low-impurity chalcopyrite—like and fahlore-type copper, possibly coming from the fahlore mines in Schwaz/Brixlegg, can result in, what they describe as, “diluted fahlore copper” or mixed (recycled) copper [96, 100]. Unfortunately, this assumption was not tested with lead isotope analyses. Nevertheless, this hypothesis appears tempting when one compares the Sb-Ag diagrams of the Alpine artefacts discussed by Grutsch and the data presented in this article. Obviously, the western and central Balkan finds from our Group 1 display a comparable pattern with objects dated to Ha A1 and earlier, clustered around a concentration of approximately 0.04% Ag and 0.17% Sb, whereas the silver and antimony concentration of the younger artefacts show a clear trend for increased values (S1 Table, Figs 6–8). Their Ag concentrations lie well in between Grutsch´s analytical definition of “diluted fahlore copper” of 0.1–0.5% [100].

Within the current state of research, it is unclear whether the elevated Ag and Sb concentrations in the Balkan artefacts are caused by a change of the ore basis, the smelting of copper ores with additional minerals or the use of mixed “diluted” copper. Each option has pro and contra arguments. Nevertheless, a change in the ore from chalcopyrite to fahlore can largely be ruled out as the concentrations of the characteristic trace elements (e.g. Sb, Ag and As) are not as high as those usually expected when smelting fahlores [100].

The second option, the smelting of ores with accessory minerals, is certainly a possibility since such ore deposits are present in the identified copper source regions in the Alps [82, 101, 102]. According to the description, the ore mineralization in the southern Alps comprises chalcopyrite and fahlore dominated ore deposits, as well as polymetallic ore veins [82]. Some of them, especially the tetrahedrite dominated ores, would appear to be a very attractive copper source with the described elevated Ag concentrations, in this respect one can point out the ore deposits from the Pfundererberg, and, Maso Erdemolo which contain (Cu)–Sb-Ag, Ag- and Bi-Ag sulfides. At least the ore deposits with additional Bi-Ag minerals can be excluded, as this would result in a metal with significantly elevated Bi concentrations, which was not observed in our case. The mean values of Bi remain low falling between 0.01–0.0172% in all three of our sampling groups.

The metal finds from the area of North Italy provide additional hints and a deeper insight into this phenomenon. Within a recent study, Caterina Canovaro and co-authors examined two hoards [103]. One of them, the Cervignano hoard, (BA D–Ha A1), contains an ingot (Cer-PS 64) with 1.9% Sb, whilst the other artefacts do not show such elevated trace element concentrations. Based on these results, they interpret this ingot as a mixture of chalcopyrite, sphalerite, galena and tetrahedrite (Cu,Fe_12_Sb_4_S_13_), like examples found in chalcopyrite from Calceranica/Vetriolo, and tetrahedrite from the Carnia or from Northern Tyrol [87, 103]. Such mixing resulted in elevated trace elements, which is comparable to the pattern observed in our study. Of further importance are analyses of the metal objects discovered at the site Frattesina in the Po valley, dated to Ha A2–Ha B1 [104] and as such, comparable with our results. All of these pieces were made of copper from the Trentino area [20, 105, 106]. In general, their trace element concentrations are comparable to the artefacts from the western and central Balkans, even though the Ni concentrations are slightly lower. Additionally, as demonstrated by the Ag-Ni and Sb-Ag diagram (Fig 8), the silver values resemble those from the two younger chronological phases of our study (Group 2 and 3). However, we also acknowledge that the state of research for the periods Ha A2–Ha B3 in Northern Italy still lacks extensive mining, archaeological and archaeometallurgical data.

The last option–the local production and use of „diluted”fahlore is also not conclusive at the current state of research—as we are lacking of comparative data of ores and ingots produced from them. The fact, that we unfortunately have no ingots dated to Ha A2 and younger in our dataset makes it impossible to theorize whether the metal was locally mixed, resulting in “diluted” fahlore copper.

We should also not exclude local copper ore deposits in Bosnia-Herzegovina as a potential source but the lack of thorough geochemical analyses and traces of the Bronze Age mining and smelting activities makes it difficult to establish any further hypothesis. There are various ore deposits with fahlore and subordinate chalcopyrite such Maškara and Slatina near Rama or Sinjakovo near Jajce that could be considered as potential sources [107] and the archaeometallurgical analyses of these ores are already in progress. If these ores, or metal smelted from them, were mixed, the obtained metal could have an altered “diluted” fahlore signature. This does not rule out the possibility that the “silver-rich” copper used in the western and central Balkans from Ha A2 onwards was smelted or melted together in its source region (e.g. in North Italy) and then traded to the Balkans.

The Slovenian territory, which connects the Alpine area with the Balkan Peninsula, offers far better options for comparison. Neva Trampuž-Orel has conducted intensive archaeometallurgical research on a large number of hoards [5, 34]. The hoards from Čermožiše, Debeli Vrh, Pekel, H. Pohorje and some pieces from Mušja jama/Fliegenhöhle form a first group dated to Ha A1. Their silver concentrations lie below the detection limit of the analytical device or vary between 0.04–0.05%. The second group consists of the hoards from Kanalski Vrh I, Šempeter dated to more recent periods (Ha B) and remaining finds from the Mušja jama//Fliegenhöhle, which again have elevated silver concentrations up to 1% and their antimony values also follow this pattern. Indeed, the younger the hoards are dated, the higher their Sb concentrations (up to 7.25%). Some of the examined finds from Slovenia that would be highly interesting from an analytical point of view (e.g. the Veliki Otok I hoard), are however not suitable for a comparison as they include objects, namely cast ingots, which are not chronologically significant. Based on their analyses, Neva Trampuž-Orel and her colleagues were able to define a “chemical” chronology for the finds from the Mušja jama/Fliegenhöhle where it appears that the concentrations of impurities can also be used for a chronological determination of objects [5]. In this work, they presented artefacts dated to Ha A1–A2 with impurities of up to 2%, whilst the finds dated to Ha B1 or younger have impurity concentrations above this level. This generally falls in line with the impurity patterns of the western and central Balkan artefacts (Fig 8). This outcome is especially important since many of the objects from this votive cave were deformed and/or burned making the typological determination almost impossible.

The studies by N. Trampuž-Orel on Slovenian objectsprovided further suggestions about the flow of raw metal, with the examination of plano-convex and so-called biconical or pick-ingots. The latter evolved in the southern Alps and then spread to various regions like France, Switzerland, Northern Italy, the Adria and to the Slovenian territory [108–110]. It is apparent that the metal from the Alps entered the Balkan Peninsula in the form of these ingots. Yet the pick-ingots are found only in Slovenia and the current research does not highlight their distribution further to the south or southeast in the Balkans. In the Kanalski Vrh I, Veliki Otok and Dragomelj hoards, the pick-ingots occur in large numbers [34]. In Trampuž-Orels opinion, the high concentration of impurities, especially in those coming from Kanalski Vrh I and Dragomelj, speak for the smelting of fahlore and copper ores with additional minerals. As copper ore deposits with elevated cobalt and nickel concentrations are unknown in the Slovenian Alps, the assumption was made that the Austrian ore deposits in the central and eastern Alpine Greywacke Zone (in the vicinity of the Mitterberg-Hochkönig,Schladming and Liezen) are likely the source for this copper, with the Zinkwand-Vöttern-Giglach area being a second possible source region [101, 102, 111, 112]. In conclusion, the trace element pattern of analyzed objects from Slovenia follow the trend of elevated Sb and Ag concentrations in European LBA artefacts already described. Additionally, one can observe that in the Slovenian territory, metal objects with higher antimony and silver concentrations (compared to western and central Balkan artefacts) are present. Furthermore, the analyses of the Slovenian ingots allows us to hypothesize that their copper also comes from the deposits in the eastern Alps as was the case for some of ingots found in the central and western Balkans. Finally, the trend from Ha B1 onwards for metal finds from Slovenia to have a high percentage of intentionally alloyed lead (up to 58% in some objects) does not apply to our dataset.

### Alloying practices—tin concentration

The re-use of fahlore-based copper starts at around 1100 BC, a time during which a decrease in tin can be detected in bronze artefacts. As the addition of fahlore copper converts the reddish chalcopyrite copper to a yellow bronze like appearance, it is assumed that the metallurgists were preferably using fahlore-copper as a raw material in the case that tin was sparse [22, 99, 113]. In the following, we will shortly address the tin concentration in the LBA objects presented here in order to verify if the observations about tin content in contemporary finds from surrounding regions also apply to our dataset.

The oldest analysed copper-based Bronze Age artefacts in the region date to BA A and have a chemical composition that still resembles the metallurgical traditions of the Copper Age with unalloyed or arsenical copper [23, 29, 30]. In the subsequent chronological stage BA A2–B1, all thus far analyzed artefacts are made of tin bronze [13, 29]. As previous studies have demonstrated, the tin concentration in the objects from the western Balkans (5.2–8.6% Sn) is somewhat higher than in the central Balkans (2.7–5.3% Sn) during this stage [13, 29]. The analyzed Middle Bronze Age objects (BA B2–BA C) commonly have a higher percentage of tin in both the western (6.0–8.8%) and central (3.8–10.1%) Balkans [13].

Among the LBA objects analyzed within this study, the tin varies between 0.002% (the detection limit of the analytical device) and 14.9% (S1 Table). Most of the unalloyed pieces can be classified as ingots or raw material, but there is also a group of finished objects having 1–8% tin. The remaining artefacts can be characterized as well-alloyed tin bronzes (8–14.9% Sn). Regarding the sampling groups, objects from Group 1 have an average tin concentration of 6.6% (Table 2). This value only marginally decreased in the following stages (Group 2 with 5.9% and Group 3 with 6.3%) with the remark that the lower average for Group 2 is caused by a single socketed axe made of unalloyed copper (Fig 3: 6, VIAS lab. no. SJLM 89). Without the axe, the average for Group 2 would rise to 6.3%.

**Table 2:** Mean and median values for various element concentrations of the artefacts under study.

element	Group 1 (n = 15)		Group 2 (n = 15)		Group 3 (n = 15)	
	mean	median	mean	median	mean	median
**Ag**	0.039	0.037	0.145	0.138	0.108	0.113
**As**	0.333	0.225	0.465	0.425	0.259	0.244
**Bi**	0.010	0.010	0.017	0.016	0.014	0.013
**Ni**	0.33	0.35	0.39	0.43	0.25	0.23
**Pb**	0.41	0.17	0.392	0.251	0.950	1.027
**Sb**	0.29	0.14	0.526	0.386	0.304	0.372
**Sn**	6.6	6.9	5.9	4.8	6.3	6.4
**Ʃ(As, Ni, Sb)**	0.95	-	1.38	-	0.81	-

The table presentss the mean and median values for various element concentrations of the artefacts under study. All values are given in mass percent.

In terms of the relationship between object groups and tin amount, our results are, to a certain extent comparable with those of previous studies in Slovenia [5, 34], Serbia [35] and in the Carpathian Basin [114]. However, as the analyses of the finds dated between the 11^th^ and 9^th^ century BC are very rare in the entire Balkans, the tin concentrations of the metal objects from our sampling groups 2 and 3 provide the first orientation about the alloying practices during this time in the study region.

The three analyzed sickles from Group 1 (Fig 2: 13) and Group 2 (Fig 3: 8–9) from Futog (4.7%) and from Drenov Do (4% and 4.8%) are in line with the assumptions about the existence of a specific bronze alloy with around 5% tin, used primarily for sickle production [34, 114]. The two spearheads from Futog (9.1%) and Novigrad (7.4%) from our Group 1 (Fig 2: 8–9) confirm the generally higher concentration of tin in spearheads dated to BA D–Ha A1 (between 8% and 9%), which is already observed in neighbouring regions [34, 35, 114]. At this point, it is difficult to estimate if the lower concentration of tin in two younger spearheads (Ha A2–Ha B1) from Brezovo Polje (6.9%) and Vojilovo (3.9%) (Fig 3:10–11) represents a general regional trend of declining tin, since comparable data is lacking.

The concentration of the tin in swords from Topolnica (7.4% and 8.4%), Antonići (8.1%), Radaljska Ada (7.4%) and Leskovo (9.1%), all belonging to the widely distributed BA D–Ha A1 archaeological types (Fig 2: 1–4; Fig 3:3), resembles the tin values in swords of this time from the Carpathian Basin and Slovenia with an average around 8% [34, 114]. Interestingly, the two flange hilted swords from the same period assigned to more local types from Kličevac (5.6%) and Trlić (5.2%) both have markedly lower amount of tin (Fig 2: 5–6). The lower tin concentration in the younger swords from Ha A2–Ha B1 from Sivec (6.3%) and Vojskova (5.7%) (Fig 3: 1–2) and especially in two weapons from Group 3 from Ilijaš (5.4%) and Pakline (4.5%) (Fig 4: 1–2) indicate that less and less tin was added in the metal alloy for swords over time. However, the absence of comparing data from the surrounding regions concerning the swords dated to Ha B1 and Ha B3 does not allow the final assessment in this regard.

The analyzed socketed axes from the western and central Balkans expose significant variations of tin in the alloy, which could be related to their multifunctional purpose. In particular, the axes from Group 2 (Fig 3: 4–7) show a great discrepancy with finds from Brezovo Polje (12.4% and 12%), Leskovo (8.5%) and the piece from Drenov Do that has almost no tin at all (0.16%). Similar tendencies are also attested for the contemporary socketed axes from Slovenia (Ha A2–Ha B1) [34]. The tin concentrations in finds from Prozor (1.2%) and Grapska (7.5%) from our Group 3 (Fig 4: 4–5) are the first hint that major differences of tin concentrations in the metal for the axes were also present in the subsequent period (Ha B3).

The tin content in the analyzed jewelry items from Groups 1 and 2 shows large deviations that could indicate diverse alloying practices on the regional and local scale. The pin from Futog has, for example, the highest tin concentration (10.1%) among all 15 finds from Group 1 (Fig 2:15), while the fibula from Demir Kapija (1.85%) from Group 2 (Fig 3:12) is among the objects with the lowest tin concertation in the presented sample set. The jewelry from Group 3 however, has a higher and more standardized tin concentration. The most of the analyzed bow fibulae with triangular footplate (Fig 4: 6–10), neck rings, bracelets and pins are all made of bronze with a tin concertation varying between 6.1% and 8.7% (S1 Table).

In summary, the tin content in the analyzed objects from western and central Balkans dated in Ba D–Ha A1 is generally in line with results from adjacent regions of Slovenia and the Carpathian Basin. The existence of specific alloys for specific objects, as testified by D. Liversage for the Carpathian Basin and by N. Trampuž Orel for finds from Slovenia, indicates the existence of metallurgical traditions that obviously also influenced most of our study area. Interestingly, few objects from Group 1, which are described as more local types (e.g. swords from Trlić and Kličevac) deviate from the standard, suggesting perhaps the presence of local workshops and new alloy mixtures with less tin.

The tin values of the artifacts from Group 2 are characterized by a great variability with objects containing either very high or very low tin concentrations. Comparable amplitudes are also observed in Slovenia [34]. The occurrence of alloys with varying tin amounts and without clear patterning in relation to object categories indicates a shift in production, with the increased development of workshops operating locally. The reason for the uneven tin amounts can be sought in the breakup of older tin supply networks that consequently led to different alloy experiments.

Finally, the tin values in the objects from Group 3 suggest a certain consolidation and a rise of new production norms with strictly locally distributed bronze types. Significant in our study, is the decline of tin in weapons and more or less standard higher amounts of tin in prestige jewelry items. Since the comparable data for this period from the adjacent territories is not available, it is currently difficult to assess whether this is a general trend or just indicative of the western Balkans where most of the finds in Group 3 come from.

## Conclusion

The presented analyses have enabled us to draw the first conclusive picture about the metal exchange systems in the western and central Balkans during the LBA (ca. 1400 to 900 BC). With regard to the copper raw materials, it is somewhat surprising that the regional ore deposits, for which geochemical data are available, can almost certainly be excluded as a potential source. Even the copper from the large deposits in eastern Serbia that was smelted during the Early and Middle Bronze Age do not seem to be relevant for the Late Bronze Age [13]. Instead, our results revealed that from 1600/1500 BC onwards, the copper from the southern Alps from the Trentino region became a main metal source for almost the entire area of the western and central Balkans. As pointed out in previous studies, the mining and smelting areas in North Italy had a dominant supply role for the whole of Italy and parts of the Eastern Mediterranean during the Late Bronze Age [105, 106, 115]. Recent studied have also demonstrated that copper from these regions was traded even to North Europe [28, 116].

In addition to the observations we have presented, the analytical results testify that the eastern Alpine ore deposits e.g. of the Hochkönig–Mitterberg mining areas were also source areas for metal exchange networks of the Balkan region [13]. N. Trampuž-Orel and B. Orel have already argued for the supply of copper from these regions, or more precisely from central and eastern Greywacke zone, into the Late Bronze Age networks in Slovenia and beyond [102]. The copper from the eastern Alps was, beside plano-convex ingots, also traded in the form so-called pick-ingots that evolved in the region between North Italy and Slovenia. However, in the western and central Balkans, the copper from the eastern Alpine area was present only in the form of plano-convex ingots, as distribution of the pick-ingots in the regions south of Slovenia is thus far unknown. The extent of the exchange networks that brought the raw material to the Balkans is also demonstrated by the Rudnik hoard in Serbia where ingots deriving from both–the southern Alps and the eastern Alps a–were identified.

In addition to the dominant copper from North Italy and from eastern Alps in Austria there is a small number of objects, especially from North Macedonia, that indicate raw material sources from some other area that is currently unknown, whilst there are also exceptional cases for the use of Mediterranean copper from Cyprus in our study area [90]. These results demonstrate that this part of the Balkans was at least marginally involved in Aegean exchange networks that reached regions of Bulgaria [33] or Sardinia [117].

A further significant outcome of the analyses we have presented is that the trace element composition of artefacts dated to Ba D-Ha A1 with some exceptions points to a chalcopyritic ore basis, whilst from Ha A2 onwards almost all finds have elevated silver and antimony concentrations that signify the input of fahlore-based copper. A comparable trend, which is undoubtedly connected with the reuse of the fahlore copper during the advanced stages of Late Bronze Age, was already observed in the Alpine region, Southern Bavaria, Sweden, Slovenia, England, France, Switzerland, Bohemia, Slovenia and Hungary [19, 21]. Thus, this metallurgical phenomenon appears to be applicable for a number of European regions. In terms of chronology, the new alloys with more impurities started to appear from approximately 1100 BC onwards and are interconnected with the unstable tin supply and the depletion of the chalcopyritic ore deposits e.g. in the Alpine regions. Another indicator of modification in the technological traditions during the phase Ha A2–Ha B1 is a strong oscillation of the tin amount as compared to more or less standardized values from the previous period. Finally, profound changes during this period are also seen in the occurrence of many new weapon and jewelry types with a local distribution in different regions of the Balkans.

Given that a number of archaeometallurgical and archaeological aspects speak for a substantial change in the raw metals used from Ha A2 onwards, it is highly remarkable that, according to geochemical analyses, the deposits in the southern Alps remained a dominant source of copper for the great majority of artefacts from the study area. The local workshops apparently still received raw material from the same areas, and the same supply network continued to persist during the Ha B3 period, with most of the repertoire being made of strictly local forms.

Considering the bronze objects from all sampling groups and, with the few exceptions mentioned above, there are no differences in terms of raw material source between regional and supra-regional types. Nearly all are made of copper from the southern Alps. This means that the same exchange network provided copper for the producers of international bronzes, wherever they might be situated, and for the local workshops, which were engaged in the founding of specific regional types. Such intensive connection between the study area and North Italy, when it comes to copper supply over a significant time span, cannot be attested in other parts of the archaeological record. Due to the geography, the close ties between Italy and the Adriatic during the Middle and Late Bronze Age are well recognizable not only in terms of metal repertoire but also in ceramic forms and decorations [118, 119]. Yet in the other regions of the Balkans, the connection with North Italy can be assumed only by few indicative metal types that are not a part of a local spectrum such is for instance a sampled pin from Group 3 (Fig 4: 15). Hence based only on archaeological finds it would be hardly possible to consider the scenario that is now strongly suggested by the analytic results.

In summary, based on our analyses, we can state that copper from ore regions in southern and eastern Alps dominated the metal supply networks of the central and western Balkans from the Middle Bronze Age and throughout o the entire Late Bronze Age. What is further significant is the fact that all regional copper ore deposits for which we have geochemical data at hand can be largely excluded as a potential source. Finally, this research marks the Balkan Peninsula as a major recipient of copper from the southern Alps and sheds a new light on the exchange and trading systems in Southeastern Europe.

## Supporting information

S1 Table Chemical composition of the analyzed objects as determined with energy-dispersive XRF. All values are given in mass percent. Se was below the detection limit of 0.01%, Te below 0.005% in all samples.
(XLSX)

S2 Table Lead isotope ratios in the samples discussed within this article. The precision of measurement is less than ± 0.01% for ratios with 206Pb in the denominator and up to ± 0.03% for 206Pb/204Pb. Definition of the ore districts and metallogenic units after [19, 82].
(XLSX)
