# Correction: Emergence of monopoly–Copper exchange networks during the Late Bronze Age in the western and central Balkans

**DOI:** 10.1371/journal.pone.0333384

**Published:** 2025-09-25

**Authors:** Mario Gavranović, Mathias Mehofer, Aleksandar Kapuran, Jovan Koledin, Jovan Mitrović, Aleksandra Papazovska, Andrijana Pravidur, Aca Đorđević, Dragan Jacanović

An additional affiliation is missing for the second author. Mathias Mehofer is also affiliated with the Human Evolution and Archaeological Sciences, University Vienna, Vienna, Austria.

The eighth author’s name is spelled incorrectly. The correct name is: Aca Đorđević.

There are errors in the Author Contributions. The correct contributions are:

**Conceptualization:** Mario Gavranović

**Data curation:** Mathias Mehofer

**Formal analyses:** Mario Gavranović, Mathias Mehofer, Aleksandar Kapuran, Jovan Koledin, Jovan Mitrovic, Aleksandra Papazovska, Andrijana Pravidur, Aca Đorđević, Dragan Jacanović

**Investigation:** Mario Gavranović, Mathias Mehofer

**Methodology:** Mario Gavranović, Mathias Mehofer

**Project administration:** Mario Gavranović, Mathias Mehofer

**Resources:** Mario Gavranović, Mathias Mehofer

**Supervision:** Mario Gavranović, Mathias Mehofer

**Writing – original draft:** Mario Gavranović, Mathias Mehofer,

**Writing – review and editing:** Mario Gavranović, Mathias Mehofer

**Writing of sections**:

Mario Gavranović: Introduction, Cultural context and dating, Materials, Results, Questions and objectives, Alloying practices, Conclusion

Mathias Mehofer: Questions and objectives, Methods, Results, Lead isotope analyses, Provenance studies and exchange networks, Trace element analyses, Copper-types, Conclusion
